# Barriers and enablers of breastfeeding in mother–newborn dyads in institutional settings during the COVID-19 pandemic: A qualitative study across seven government hospitals of Delhi, India

**DOI:** 10.3389/fnut.2022.1052340

**Published:** 2022-12-08

**Authors:** Arti Maria, Ritika Mukherjee, Swati Upadhyay, Kumari Pratima, Tapas Bandyopadhyay, Rachita Gupta, Bhawna Dubey, Abhinav Sharma, Pranaya Kumar Mall, Manaswinee Sahoo, Keshav Kumar Pathak, Priyanka Pawar, Archisman Mohapatra

**Affiliations:** ^1^Department of Neonatology, Atal Bihari Vajpayee Institute of Medical Sciences and Dr. Ram Manohar Lohia Hospital, New Delhi, India; ^2^Generating Research Insights for Development (GRID) Council, Executive Office, Noida, Uttar Pradesh, India; ^3^WHO Country Office for India, Nutrition, R.K. Khanna Stadium, Safdarjung Enclave, New Delhi, India

**Keywords:** breastfeeding, newborn care, India, COVID-19, hospital, isolation, baby friendly hospital initiative, family centered care

## Abstract

**Introduction:**

The COVID-19 pandemic disrupted newborn care and breastfeeding practices across most healthcare facilities. We undertook this study to explore the barriers and enablers for newborn care and breastfeeding practices in hospitals in Delhi, India for recently delivered mother (RDM)–newborn dyads during the first wave of the COVID-19 pandemic (2020) and inductively design a “pathway of impaction” for informing mitigatory initiatives during the current and future pandemics, at least in the initial months.

**Materials and methods:**

We used an exploratory descriptive design (qualitative research method) and collected information from seven leading public health facilities in Delhi, India. We conducted separate interviews with the head and senior faculty from the Departments of Pediatrics/Neonatology (*n* = 12) and Obstetrics (*n* = 7), resident doctors (*n* = 14), nurses (labor room/maternity ward; *n* = 13), and RDMs (*n* = 45) across three profiles: (a) COVID-19-negative RDM with healthy newborn (*n* = 18), (b) COVID-19-positive RDM with healthy newborn (*n* = 19), and (c) COVID-19 positive RDM with sick newborn needing intensive care (*n* = 8) along with their care-giving family members (*n* = 39). We analyzed the data using grounded theory as the method and phenomenology as the philosophy of our research.

**Results:**

Anxiety among clients and providers, evolving evidence and advisories, separation of the COVID-positive RDM from her newborn at birth, providers' tendency to minimize contact duration and frequency with COVID-positive mothers, compromised counseling on breastfeeding, logistic difficulties in expression and transportation of COVID-positive mother's milk to her baby in the nursery, COVID restrictions, staff shortage and unavailable family support in wards and nursery, and inadequate infrastructure were identified as major barriers. Keeping the RDM–newborn together, harmonization of standard operating procedures between professional associations and within and between departments, strategic mobilization of resources, optimization of human resources, strengthening client–provider interaction, risk triaging, leveraging technology, and leadership-in-crisis-situations were notable enablers.

**Conclusion:**

The separation of the RDM and newborn led to a cascade of disruptions to newborn care and breastfeeding practices in the study institutions. Separating the newborn from the mother should be avoided during public health emergencies unless there is robust evidence favoring the same; routine institutional practices should be family centered.

## Introduction

Feeding mother's own milk (MOM) is advantageous for the recently delivered mother (RDM)–newborn dyad (1–6). Governments worldwide, care providers and breastfeeding support groups, the WHO, and several other stakeholders have been working toward its universalization. It is recommended that mothers should initiate breastfeeding immediately after birth, provide exclusive breastfeeding for up to 6 months, and continue breastfeeding along with complementary for at least up to 2 years. However, ensuring optimal breastfeeding practices among the population at large has been an uphill task for countries, especially low- and middle-income countries (LMICs). Institutional practices around birth and immediately thereafter, e.g., early-uninterrupted skin-to-skin contact, early initiation of breastfeeding, rooming-in, involving families in care of RDM–newborns, help in establishing and sustaining optimal newborn care, and breastfeeding practices (7–9). The increasing rate of institutional deliveries across the world provides an opportunity to ensure that optimal newborn care and breastfeeding practices are reinforced for mother–newborn dyads before their discharge. Unfortunately, the COVID-19 pandemic has challenged health systems worldwide leading to the disruption of routine healthcare services. Anticipating the pandemic's disruptive effect on RDM–newborn care services and optimal breastfeeding practices (10–13), the WHO released mitigatory advisories in early 2020 (14–16). Professional associations and institutional authorities also made efforts to mitigate the disruptions. Still, breastfeeding practices got compromised even in the most advanced healthcare setups (17, 18). Studies published later into the pandemic, i.e., in 2021 and 2022 indicate that breastfeeding practices got compromised in newborns during the pandemic due to a gamut of reasons including lack of high-quality professional services, peer-to-peer support, and social vulnerability and ignorance amidst challenges posed by the mass movement restrictions (19–21).

India has the world's largest annual birth cohort (~25 million) (22). Nevertheless, it continues to struggle in ensuring exclusive breastfeeding (EBF) in infants (as per the latest Demographic and Health Survey in India (NFHS-5), EBF rates were only about 63.7%) India promotes breastfeeding for improving newborn survival and development (23–25). Hence, India prioritizes institutional deliveries and baby-friendly hospital initiatives (BFHI) and has witnessed appreciable improvement in institutional practices for maternal and newborn care. In the initial months of the pandemic, India had to suspend non-essential healthcare services, e.g., out-patient consultations and elective procedures so that health care resources could be mobilized and redirected to pandemic combat (26). Teaching medical institutions (the front-leaders of optimal evidence-based practices) also had to reposition their resources to meet surge demands (27). During these initial months, India also witnessed a dip in institutional deliveries, duration of stay in the hospital post-delivery, and post-partum follow-up (both for the mother and the newborn) (28). To mitigate the potential disruption caused by the pandemic on institutional care of mother–newborn dyads and to standardize practices across India, the Federation of Obstetric and Gynecological Societies of India (FOGSI), National Neonatology Forum of India (NNF), and Indian Academy of Pediatrics released a joint statement in April 2020 (29). Nevertheless, newborn care and optimal breastfeeding practices likely got affected adversely during the pandemic. While there was an inadequate understanding of the “pathways” through which the COVID-19 pandemic impacted newborn care and optimal breastfeeding practices in institutional settings in India during the initial months of the pandemic, there was a gap in knowledge on how these could have been best salvaged. As with the guidance documents of WHO, even the joint statement needed contextual adaptation for implementation across healthcare facilities which most premier health care facilities would have likely undertaken. Thus, there was a need to explore, identify, and learn from experiences and practices across institutions. In the wake of the impediments caused by the COVID-19 pandemic, there was also a window of opportunity to examine institutional practices for newborn care and breastfeeding for critical learnings for building resilient “BFHI” systems. Consequently, we undertook this study to explore the barriers and enablers for newborn care and breastfeeding practices in hospitals in Delhi, India, for recently delivered mother (RDM)–newborn dyads during the first wave of the COVID-19 pandemic (2020) and inductively design a “pathway of impaction” for informing mitigatory initiatives during the current and future pandemics, at least in the initial months. Thus, in this study, we explored how newborn care and breastfeeding practices got impacted in institutionalized mother–newborn dyads in the major teaching medical institutions in Delhi, India in the initial months of the pandemic. We also explored what challenges these institutions faced in their effort to mitigate risk and sustain services and what innovations/enablers they identified in the process that could help inform ongoing practices and also develop standard protocols for future disasters. We believe that policy and program managers and healthcare providers engaged in mother-and-baby care and breastfeeding promotion efforts will find the findings useful for evidence-based action.

## Methods

### Study design and setting

We used an exploratory descriptive qualitative design and conducted in-depth interviews (IDIs) with participants from seven medical institutions in Delhi; six of these were premier teaching medical schools in India. Two of the institutions were operating as ‘exclusive COVID-care facilities' at the time of data collection. Delhi had been one of the first and worst affected states and union territories in India during the COVID-19 pandemic (30). It also has some of the premier teaching medical institutions that not only provide state-of-the-art health services to a huge population but also actively engage in innovating and designing case management protocols that are adapted/followed by institutions nation-wide. Exploration of the impact of the COVID-19 pandemic on newborn care and breastfeeding practices in these institutions and how these managed mitigatory efforts held the promise of identifying not only the factors and pathways of impaction but also the potential nodes for intervention. These health facilities are spread across Delhi and cater to a diverse range of clientele across socio-economic strata from the Delhi National Capital Region as well as referral patients from across India with variegated newborn care and feeding practices.

### Participants

In the study, we considered the “newborn” as the central focus (though it would have been ideal to center around mother–newborn dyads, it was operationally difficult at that point in time). We prepared a list of the stakeholders involved in newborn care and feeding in institutional settings by preparing a “pathway of care” schematic (described below in Study instruments). To start with, we decided to interview the head and the second-senior-most faculty in the departments of neonatology or pediatrics (if the institution did not have a department of neonatology) of the selected institutions. However, we also felt that we needed to get inputs from the head of the department of obstetrics at these institutions in order to triangulate information comprehensively and in line with our phenomenological philosophy. Consequently, we interacted with seven head obstetricians from the institutions studied. Neonatologists and obstetricians were responsible for the care of the newborn and the RDM, respectively, in maternity wards, isolation wards, nurseries, and newborn intensive care units. We considered these HCPs as a common group for triangulating information from “administrative leadership” perspective. Having three such HCPs from an institution added up to a total of 21 respondents in this category and were deemed adequate to saturate information. However, our team also appreciated that most of the maternal–newborn care, and especially that related to breastfeeding, in these institutions was delivered through resident doctors and nurses posted in the labor room and maternity wards. Hence, we requested the neonatology/pediatrics heads to nominate two residents and two nurses from their teams who had been posted in the care of RDM newborns for the longest duration and among the ones most actively involved in caregiving to this clientele among their colleagues. We requested to identify those residents and nurses who had been working in the institution before the pandemic hit, i.e., before February 2020. Thus, at each center, we selected these participants purposively. At that point, we assumed that with as many resident and nurse participants, we would most likely hit information saturation for each (31).

At each center, we included RDMs across each of the three profiles: (a) COVID-19-negative RDM with a healthy newborn, (b) COVID-19-positive RDMs with healthy newborns, and (c) COVID-19-positive RDMs with sick newborns. We did not intend to stratify our results according to the RDM–newborn profile since we aimed at characterizing the “phenomenon” at the institutional level; having the three profiles of RDM–newborn dyads allowed for capturing nuanced experiences and insights. We aimed at interviewing as many participants in each profile till we hit information saturation. These participants were selected purposively with the help of the neonatology residents while balancing data saturation requirements, operational feasibility, participation from the seven healthcare institutions studied, and other socio-demographic characteristics (the socio-economic class, literacy, religion, parity of the RDM, and gender of the newborn). We chose RDMs who had delivered beyond 7 days preceding the date of the interview to a maximum of up to 10 days before. We kept this day-range in order to capture institutional experiences. We also undertook non-formal interactions (NFIs) with the RDMs' escorting family members (1 per RDM) at the hospital.

We interacted with all participants with prior informed consent and appointment. We did not have any refusal to participate.

The participant profiles are shown in Table 1.

**Table 1:** Distribution of participants from the study sites (*n* = 7).

**Participant profile**	**Medical institution (study site)**∧								**Total**
		**A**	**B** ^*^	**C**	**D** ^*^	**E**	**F**	**G**	
Health care workers	Head/ Senior faculty, (Neonat./Peds.)	02	02	02	01	02	01	02	12
	Head (Obstetrics)	01	01	01	01	01	01	01	07
	Resident doctor (Neonat./Peds.)	02	02	02	02	02	02	02	14
	Nurse	02	02	02	02	02	02	01	13
	Total	07	07	07	06	07	06	06	46
Recently delivered mothers (RDMs)	COVID-negative mother with healthy baby	01	0	09	0	0	05	03	18
	COVID-positive mother with								
	Healthy newborn	03	03	02	05	03	01	02	19
	Sick newborn	01	04	03	0	0	0	0	08
	Total	05	07	14	05	03	06	05	45
Attendants	Husband	03	03	12	04	02	08	02	34
	Other family members	0	01	01	0	02	0	01	05
	Total	03	04	13	04	04	08	03	39

^**∧**^**A:** Maulana Azad Medical College and GB Pant Institute of Post Graduate Medical Education and Research (MAMC and GB Pant PGIMER), **B:** University College of Medical Sciences and Guru Teg Bahadur Hospital (UCMS and GTB), **C:** Atal Bihari Vajpayee Institute of Medical Sciences and Dr. Ram Manohar Lohia Hospital (ABVIMS and Dr RML), **D:** Deep Chand Bandhu Hospital (DCBH), **E:** Vardhman Mahavir Medical College and Safdarjung Hospital (VMMC and SJ), **F:** Lady Hardinge Medical College and Kalawati Sharan Hospital (LHMC and KSH), and **G:** All India Institute of Medical Sciences (AIIMS), New Delhi.

* Designated COVID-hospitals.

### Study instruments

For identifying the items of inquiry, we convened a meeting of the unit head and four faculty members (Neonatology), one lactation counselor, one obstetrics resident, and two extramural public health researchers with experience in qualitative methods. We prepared a “pathway of care” schematic for a pregnant woman reporting to a typical hospital in Delhi for delivery during the pandemic until her and her baby's discharge from the hospital. The pathway was reviewed with the three prototype RDM–newborn dyads (mentioned above) in mind. We anecdotally plotted the possible disruptions in pre-pandemic institutional practices at pre-delivery, at time of delivery, immediately post-delivery, during stay in the hospital, at discharge, and during follow-up. We reviewed relevant literature and designed interview guides for each participant category, pretested, and finalized them. We developed separate instruments for each participant category. Some of the items of inquiry were, however, retained across two or more tools. Supplementary Box 1 provides the tool development process. Box 1 illustrates the topics covered in the interviews and the number of items in each tool for the respective participant category. A checklist was prepared for NFI with the RDMs' escorts.

Box 1 **Topics discussed in the interviews with Unit Heads, Resident Doctors, Lactation Counselors/Nurses, and Recently Delivered Mothers (RDMs).**

**Interviews with Unit Heads**

- Overall effect of COVID-19 on breastfeeding promotive approaches
- Modifications made to accommodate IPC protocols in care of newborn
- Practices around testing of RDMs for COVID-19
- Challenges in implementing guidelines for breastfeeding and mitigating strategies
- Best practices for promoting optimal breastfeeding according to COVID-19 status
- Practices to reinforce optimal breastfeeding in RDMs after discharge
- Impact of COVID-19-related anxiety and stigma in care of RDM–newborn dyads
- Requirement for further skill building of staff
- Challenges in staff allocation for COVID duty
- Mitigation strategies for staff shortage
- Ways to ensure inter-departmental coordination while managing RDM–newborn dyads
- Suggestions for improving policies and practices

**Interviews with Resident Doctors**

- Adaptation of practices for newborn care immediately after delivering a baby
- Adaptation of routine in-patient services for RDM–newborn dyads
- Challenges experienced in advising for expression and feeding of breastmilk during COVID-19*
- Impact of COVID-19 on practices for optimal breastfeeding among RDMs*
- Challenges to breastfeeding during the pandemic and its impact on counseling strategies
- Receptivity of RDMs to breastfeeding advice during COVID-19 and its determining factors*
- Strategies adopted for managing babies born to COVID-19-positive mothers as compared to those COVID-19 negative and its effectiveness
- Challenges to decision-making with regard to breastfeeding practices
- Self-reflection on individual performance in ensuring optimal breastfeeding practices in RDMs*

**Interviews with Lactation Counselors/Nurses**

- Effect of COVID-19 in usual way of counseling of RDMs
- Challenges to effective lactational counseling during the pandemic and mitigating strategies
- Adaptation of newborn care practices for COVID-19 negative mothers
- Most effective strategies for reinforcing optimal newborn feeding practices

**Interviews with Recently Delivered Mothers**

- Experience regarding care and feeding of your newborn in the hospital
- Advice received on newborn feeding from hospital and family members during hospital stay and its impact
- Challenges experienced in feeding newborns during hospital stay and ways to address them
- Support received from the hospital that have helped her in feeding her newborn
- Support expected from hospital staff in feeding newborns during hospital stay
- Support expected for optimal feeding after discharge and in follow up

*Also discussed with Lactation Counselors/Nurses

IPC-Infection prevention and control; RDM- Recently delivered mother, COVID- Coronavirus disease

### Data collection

Six interviewers collected data—two qualitative researchers [AMo (male, MD) and RM (female, PhD)] and four neonatology faculty members [two females (SU and KP) and two males (SK and TB)], trained in interviewing techniques. The team interacted with participants over video meetings (Google Meet and WhatsApp) since physical meetings were restricted during the pandemic (32).

### Data management and analysis

IDIs with the doctors were analyzed using English transcripts. Responses from nurses, RDMs, and their escorts were directly analyzed from the audio (“tape analysis”) to allow for the comprehension of responses that were in mixed language (Hindi and Indian English) and often emotional (33, 34). Analysis was done using NVIVO (35). Responses were free-listed and coded thematically using the grounded theory approach by RM, AMo, and PP (female, MPH), in consensus (36). We achieved data saturation for each participant category (31). The code frequencies were grouped as follows: “None” (0%); “Few” (below 25%); “Some” (25–49%); “Many” (50–74%); “Most” (75–99%); and “All” (100%) (37). “Quotable quotes” were identified from the responses.

We improvised the “pathway to care” schematic as an inductive framework enumerating the disruptions to breastfeeding practices in institutionalized RDM–newborn dyads. For this, we (RM, AMo, PP, AMa, SU, KP, and TB) triangulated narratives across stakeholder categories and incorporated feedback from the larger team of authors between November 2020 and February 2021 through an iterative process. The findings were deemed consistent with the data.

Quantitative information was summarized as frequency and proportion using MS Excel (MS Office 365).

### Quality assurance

The RDMs and their escorts were interviewed at the hospital/home between the 7^th^ and 10^th^ day of delivery as per their convenience. We conducted and audio-recorded each interaction with a prior appointment and informed verbal consent for participation in the interview and for audio-recording, which was also recorded prior to the initiation of the interview. Written consent was difficult to obtain due to the COVID-19 physical access restrictions in the isolation wards and healthcare facilities. The interactions were done in the participant's preferred language. The HCPs were interviewed in the office. The IDIs were limited to 45-to-75 min, while the NFIs were for about 10–15 min each to manage respondent burden and sustain engagement. Trained interviewers administered the interview items verbatim using structured interview guides. The responses were audio-recorded on multiple devices to protect against data loss. Transcripts were matched with the recordings for accuracy and completeness before analysis; we did tape analysis to capture the “sentiments” of the respondents. Findings were reported using the COnsolidated criteria for REporting Qualitative research (COREQ) checklist.

## Results

### Participant profile

The age of the senior HCPs (neonatologists, pediatricians, and obstetricians) was 58.1 ± 3.4 years, the residents 29.3 ± 3.1 years, and nurses 43.2 ± 5.6 years. The age of the RDMs was 23.8 ± 3.2 years; 44.4% (*n* = 20) of these were first-time mothers, 82.2% (*n* = 37) were Hindus, 15.6% (*n* = 7) had 0–5 years of formal education, 8.9% (*n* = 4) were from economically weaker section, 51.1% (*n* = 23) had delivered through cesarean section. Of the newborns, 44.4% (*n* = 20) were female. We have provided the details of the RDMs' profiles and newborn care and feeding practices in Supplementary Table 1.

### Perceived barriers to optimal breastfeeding and newborn care practices

Here, we have narrated the key barriers across RDM–newborn profiles (Table 2); these were most prominent in the initial months of the pandemic.

**Table 2:** List of core themes and sub-themes for optimal breastfeeding practices emerging from inductive data analysis and their code prevalence according to the different groups of recently delivered mothers.

**Core theme**	**Sub-themes**	**Code prevalence** ^*^			
		**Healthy (COVID-19 -ve) RDM with healthy newborn (*N* = 18)**	**RDM with COVID-19 and**		**Total** **(*N* = 45)**
			**Healthy newborn (*N* = 19)**	**Sick newborn (*N* = 8)**	
Perceived barriers	Compromised newborn care	Many	Most	All	Most
	Anxiety related to COVID-19	Many	Many	Some	Many
	Difficulty in breastfeeding or expressing breastmilk	Many	Many	Many	Many
	Inadequate counseling on IPC for newborn care	Many	Few	Some	Some
	Inadequate knowledge on breastfeeding	Some	Few	Many	Some
	Management and logistic issues	Some	Some	Many	Some
	Delayed or unsatisfactory inpatient care services	Some	Some	Some	Some
	Rooming in avoided	Few	Some	All	Some
	Mother and newborn were discharged separately	Few	Some	Few	Few
	RDMs lacked support as attendants entry into the wards was restricted	Few	Many	Some	Some
	Problems faced during admission for delivery	Few	None		Few
	Inadequate/ lack of support with follow-up care	Few	Few		Few
	Lack of privacy for RDMs		Few		Few
	Referral transfer between hospitals for COVID		Some	Many	Few
Perceived enablers	Counseling on breastfeeding	Most	Many	Most	Many
	Counseling on COVID-19 to allay fear	Few	Many	Some	Some
	Optimizing time for discharge from hospital for mother–newborn dyads	Few	Few	Few	Few
	Infrastructural modifications and “zoning” during COVID	Few	Some	Few	Some
	Additional measures were undertaken for control of infection and anxiety among patients and providers	Many	Few	Some	Some
	No help required to the RDM in feeding the newborn	Many	Some	Many	Some
	Support from family for follow-up care	Some	Some	Some	Some
	Support from family members during hospital stay	Some	Some	None	Some
	Support from hospital and doctor for follow-up treatment	Some	Some	Many	Some
	Support received in inpatient care	Many	Most	Most	Most
	Received comprehensive advice on newborn care & infection control practices	Many	Most	Most	Many
	Family members advised RDM to breastfeed		Few	Many	Few
	Infrastructural modifications for better support		Some		Few
	Support to RDMs during isolation		Some	Many	Some
	Support from the hospital staff with feeding of the baby		Some	Most	Some
	No help with follow-up care required			Few	Few
	Prompt care of the newborn received			Many	Few

* Percentages grouped as: “None (0%); Few (below 25%); Some (25–49%); Many (50–74%); Most (74–99%); All (100%)”.

HOD, heads of department/unit of Neonatology, Pediatrics, Obstetrics; RDM, Recently Delivered Mother, COVID, Coronavirus disease.

#### Anxiety related to COVID-19

Providers as well as clients were anxious that they might contract and/or transmit SARS-CoV-2 with fatal outcomes. Staff anxiety was attributable to inadequate availability and knowledge of using personal protective equipment (PPE), and unavoidable interactions with patients and escorts who often lacked COVID-appropriate behavior. Several staff members got exposed inadvertently, especially during posting in COVID-suspect wards, and had to undergo quarantine; this heightened anxiety and reluctance among colleagues.

“Reluctance was from seniors, including me. But residents and sisters were always prompt and discharged their duties sincerely, not putting senior people at risk of infection for work which may be handled (over phone) without going (physically).” (HOD, Neonatology)

During the initial months of the pandemic, institutional isolation had been made mandatory for all individuals testing positive for SARS-CoV-2. The RDMs, especially those asymptomatic, found such isolation extremely distressful. They were often not convinced that they were infected and did not want to co-isolate with those with symptomatic infection.

“I did not have fever or cold. I had no symptoms, not even breathlessness. I had absolutely no problems. Even then I was told that I am corona positive. This is my exact problem here. How can they do that? I still have no symptoms and no ailments. No cough, no cold, no fever. Yet, they say I am corona positive.” (-RDM, COVID-19 positive with healthy baby)

There were instances when RDMs, including those negative for COVID-19, resisted rooming-in and/or breastfeeding, fearing transmission of infection to their newborns. There were also instances when RDMs were more willing to breastfeed than to express and transport; they felt that storage may lead to an increased risk of infection. Clients feared that they and/or the newborn may get infected during the hospital stay. At times, family members did not want to accompany the RDM to the hospital for fear of getting infected. The RDMs and family members would often insist on discharge immediately after delivery. Family members also resisted rooming-in, breastfeeding, and transportation of expressed milk citing the risk of transmission of infection.

“I still feel that my baby became COVID positive because I breastfed. I hope I did not infect him, but I feel so because I breastfed him. So, I do not want to take any further risks. I do not want to breastfeed him again.” (-RDM, COVID-19 positive with sick baby)

#### Logistics and operations challenge

As separate wards had to be created for risk stratification, resources often fell short. Procurement of new equipment had to be expedited and prudently distributed between the wards. PPEs, being in short supply, had to be used frugally. There was a lack of space for expansion. In most places, the isolation ward for the RDM was relatively far off from the nursery making transportation of expressed milk difficult. There were times when suspected COVID-19 patients and non-COVID-19 patients had to be kept in the same area, leading to increased anxiety among the RDMs and those accompanying them. Distancing between beds and rooming-in was challenging, and privacy could not be ensured for the RDMs to breastfeed or express milk.

#### Inconsistencies in evolving guidelines

As evidence was evolving fast, hospital and departmental protocols had to be updated frequently. The staff faced difficulty in adapting to the frequent change in the hospital protocols. Providers reported that obstetricians and neonatologists differed in respective departmental protocols for skin-to-skin contact, rooming-in, breastfeeding, and discharge. Initially, there was a lack of clarity on responsibilities and coordination between the departments.

“Problem is about the mindset and overcoming these resistances with our own *[name of department]* colleagues. I am strong minded, I get it implemented if these recommendations are strong, I still want to do it, but probably it's not all in my hands. I will definitely work on it, we as a team will try to do something and definitely, we will make it happen.” (- HOD, Department anonymized)

#### Inadequate staffing

Given the surge in adult patients due to COVID-19, staff from the neonatology as well as obstetric departments was diverted toward adult wards, leading to acute staff shortage therein. The raging pandemic, quarantine protocols, and a few staff resignations accentuate the shortage. At times, to meet surge preparedness and/or meet gaps in staff strength, personnel from other specialties who had little/no experience in maternal–newborn care were posted in nurseries and maternity wards.

“Mother-baby dyads are being handled by health care providers who are usually not taking care of the newborn or mothers. So definitely we are trying to educate more and more.” (-HOD, Neonatologist)

Delays in test results for the RDMs led to prolonged stay of the newborn in the nursery, thus overburdening the inadequate staff therein. PPEs also reduced the staff's efficiency.

“Repeated counseling, getting expressed breast milk, looking at babies frequently, that all needs manpower, and time and manpower, get doubled when you are working with PPE.” (-HOD, Neonatologist)

#### Entry restrictions

Due to infection prevention and control (IPC) protocols, RDM's escorts were disallowed in delivery rooms and maternity wards. RDMs, especially those who were primiparous or had delivered by cesarean section, needed support for self-care and expression of breastmilk and faced many difficulties; escorts were anguished.

“Because of COVID, mother and baby were completely isolated. I or any other family member was not allowed inside. But this is not right, especially because she had C-section and someone should have been allowed to take care and help her.” (- Family member of RDM)

#### Separation of mother and newborn

In the initial months of the pandemic, providers were unsure if breastfeeding was safe during COVID-19. If the RDM was COVID-19 positive or suspect, her newborn was separated immediately after the birth and started on artificial milk till the dyad tested negative for SARS-CoV-2. At times, a mother delivering at a non-COVID hospital was referred to a designated COVID hospital on being detected positive, while her baby was retained at the hospital of delivery. The separation was quite distressing for the RDMs and their families.

“I was not allowed to breastfeed or send expressed breastmilk to my baby. My baby was not given my milk. (-RDM, COVID-19 Positive with healthy baby)

“I did not even see my baby since birth, how could I have possibly breastfed her?”

(-RDM, COVID-19 Positive with healthy baby)

“I have to go to the other building to get the milk. I do it about 7-8 times a day. It is difficult as I have arthritis, but I have to do it because if I won't, who else (would do it)!” (- Mother-in-law of a COVID-19 positive RDM)

#### Compromised provider–patient interaction

Interaction between the doctor, RDMs, and family members got compromised due to fear and limited visitations in face of staff shortage. The gap for counseling services was most glaring at places where these were most needed.

“When I had my first child, I was advised well about the breastfeeding. However, this time they explained nothing to me. They did not discuss or advised anything. Since delivery, the doctor has not even visited once to check on me.” (-RDM, COVID-19 negative)

Repeated donning and doffing of PPEs were inconvenient. Furthermore, conversations with masks on their face compromised both the duration and quality of interaction. Massages for newborn care and breastfeeding could not be provided consistently due to various reasons, e.g., reallocated staff often lacked standardized skills and motivation to counsel, specialties differed in their opinions, etc. All these factors adversely affected the opportunities for counseling in the early postnatal period for establishing optimal breastfeeding. Pandemic restrictions had separated the RDM from the family. Thus, the two had to be counseled separately. This not only increased the workload of the staff but was also deemed as less efficient than counseling both together.

Some of the RDMs and family members also reported being unaware of the newborn's care and wellbeing, indicating a gap in communication.

#### Discharging RDM and newborn separately

For instances where the RDM was COVID-positive, the family resisted rooming-in of the baby and rather preferred to take the baby home. In some cases when the mother was COVID-negative and the baby required nursery care, even obstetricians as well as families preferred that the mother be discharged to minimize risk exposure. Discharging the RDM and her newborn at different time points disrupted breastfeeding practices. Families also reported difficulty in managing the newborn at home without the mother.

“I am troubled that they are not discharging my wife. I am staying with the baby alone in the house. It's difficult for me to manage. Yesterday they asked me to come at 10 am today and they would discharge, now they are saying they will not” (- Family member of RDM)

#### Inadequate follow-up care

Most hospitals had suspended post-partum as well as neonatal follow-up clinics and had moved on to patient-initiated telephonic follow-up. However, with frequently changing staff, it was difficult for an RDM to contact the staff that had tended to her and her newborn when in the hospital. Also, hospitals at times did not have phone numbers of the RDMs or their families to contact after discharge. The providers were unsure if optimal breastfeeding practices were sustained once discharged from the hospital.

#### Client dissatisfaction

While most patients were satisfied, some complained of inadequate communication during their hospital stay. Family members at times reported facing stigmatized behavior and inadequate help from the hospital support staff. The RDM in the isolation ward as well as family members felt under-informed; they reported a gap in communication with the nursery staff. As entry into the nursery was restricted, they questioned the quality of care for the newborn.

While most clients appreciated improved hygiene and IPC at the hospital, others expressed dissatisfaction. They complained about unclean wards, walkways, toilets, cumbersome administrative processes, uncomfortable beds (no backrest to support breastfeeding mothers), and lack of privacy and comfort. Family members accompanying RDMs reported overcrowding, lack of chairs in waiting areas, and lack of adequate, clean, and separate toilets for men and women.

“Hygiene was very bad. 2 maternity rooms 1 room had 12 – 15 beds while the other room had 3 beds. At one point 10 – 12 patients were there who were all using one common toilet. The toilet was being cleaned only once in 24 hrs. (There were) cockroaches in the room” (- Family member of RDM)

The clients complained that stringent IPC practices coupled with conflicting information from different health personnel led to avoidable delays in services. Family members had to arrange their own personal protective provisions adding to their out-of-pocket expenditures.

“We reached emergency at 2 am and then the report was not accepted, she was not given the private ward…These were not good. The Gynae doctor said we can do the test from outside but emergency doctor refused…Took too much of time in admission” (- Family member of RDM)

### Overcoming barriers to optimal breastfeeding and newborn care practices

We noticed temporal trends in the providers' and participants' attitudes to COVID-19 after the initial months; practices and innovations emerged as enablers (Tables 3, 4) in response to disruptions caused by the pandemic.

**Table 3:** List of core themes and sub-themes for optimal breastfeeding practices emerging from inductive data analysis and their code prevalence according to the healthcare providers.

**Core theme**	**Sub-themes**	**Code prevalence** ^*^		
		**HOD**	**Resident doctors**	**Nurses**
Perceived barriers	Anxiety related to COVID-19	All	Most	Most
	Reduced quality of provider–patient interaction	Most	Many	All
	RDMs lacked support as attendants' (family members) entry into the wards was restricted	Most	Most	Many
	Mother and newborn were discharged separately	Some	Some	
	Inadequate/ lack of support with follow-up care	Few	Some	Few
	Inadequate staff capacity	Most	Most	Many
	Separation of mother and newborn immediately after birth and its prolongation	Most	Most	Most
	Inconsistent and evolving guidelines	Most	Some	
	Inadequate infrastructure and logistics	Many	Most	Most
Perceived enablers	Additional measures were undertaken for control of infection and anxiety among patients and providers	All	Most	Many
	Optimizing time for discharge from hospital for mother–newborn dyads	Most	Many	Few
	Infrastructural modifications and “zoning” during COVID	Most	All	Most
	Guidelines coming up after initial months and their sharing and adherence by staff	Few	Some	Few
	Evolution of existing practices for newborn care and breastfeeding during COVID	Most	Most	Most
	Change in attitude of patients and healthcare workers with time	Many	Some	Many
	Leadership in promoting breastfeeding in newborn	Some	None	Few
	Good coordination and teamwork between Obstetrics and Neonatology	Most	Some	Few
	Innovation of follow-up practices	Many	Many	Some
	Staff deployment	Most	Some	
	Promotion of optimal breastfeeding in the COVID-19 pandemic	Most	Most	
	Support (physical) provided to RDMs during the isolation	Some	Few	
	Regular testing for COVID	Some		
	Able to follow newborn care practices because case load of deliveries during COVID is low	Some		
	Willingness of the RDMs to be counseled		Some	
	Choice given to the family to decide rooming in if mother is positive		Few	
	Positive receptivity of breastfeeding counseling by RDMs			Most

* Percentages grouped as: “None (0%); Few (below 25%); Some (25–49%); Many (50–74%); Most (74–99%); All (100%)”.

HOD, heads of department/ unit of Neonatology, Pediatrics, Obstetrics; RDM, Recently delivered mother, COVID, Coronavirus disease.

**Table 4:** Barriers experienced, and counter measures undertaken to ensure optimal breastfeeding among institutionalized mothers during COVID-19 pandemic.

**Perceived barriers**	**Examples of counter measures/innovations undertaken**
^*^ Inadequate client–provider interaction ^*^ Anxiety related to COVID-19 (Hesitancy of mother/ family members; Hesitancy of HCWs)	**Nursery staff were made more easily accessible to RDMs** •Centralized facility of audio and video for the family members to connect with the RDMs; mobile and video calling facility in the nursery for communication between doctor and RDM **Strategies and content for counseling of RDMs was modified in the context of COVID-19 to increase its effectiveness** •Shared picture of mothers breastfeeding with masks; counseling done individually and/or in smaller groups; other breastfeeding mothers involved in counseling of the RDMs to motivate for breastfeeding; senior doctors counseled mothers who were extremely depressed and resisted breastfeeding **Repeated counseling of family members** •Counseled to do away with anxiety and agree to breastfeeding by RDM; family members encouraged and involved in decision-making.
^*^ Mother–baby discharged early/ separately	**Optimizing time for discharge from hospital for mother**–**newborn dyads**
^*^ Reduced post-discharge follow-up	**Ensured seamless follow-up of RDMs and newborns after discharge** •Dedicated teams/ personnel and phone lines for telephonic and video calls assigned; developing robust checklist for scheduled calls for follow up
^*^ Deployment of staff not trained in pandemic-appropriate skills ^*^ Staff shortage	**Staff management optimized for non-disruption of services while maintaining staff motivation and protection**. •Rotational duties followed by quarantine; staff sensitization and training up-scaled for sustained motivation and skill enhancement; active involvement of senior staff and department leadership in the COVID ward to set an example; accommodation for HCWs with dedicated conveyance was arranged
^*^ Logistic constraints ^*^ Frugal use of PPE ^*^ Need to harmonize Neonatology-Obstetric SOPs for Maternal-Newborn Care ^*^ Infection prevention protocols in hospitals	**Infrastructural changes to meet improved IPC requirement, need for risk triaging, and for maximizing efficiency**•Reassigning and redesigning of resources to facilitate and promote optimal breastfeeding; curtains and screens were put between beds for privacy to the RDM for breastfeeding/ expression; documentation of the patients done in the duty room and not along the bedside; created antenatal OPD for COVID cases only; installation of camera in the wards/ rooms for remote monitoring of patients
	**Zoning according to risk for minimizing risk of exposure** •Zoning of nursery and maternity wards depending on the COVID status; separate red/ green zone depending on patient movement and location of wards
	**Special IPC measures for crowd management** •Screening of accompanying family members for COVID-19 symptoms before allowing entry; sanitizer dispensers were installed at places; crowd management SOPs were developed
	**Support mobilized for RDMs – both logistic and emotional** •Attendants allowed and arrangements were made for attendants to help mother with all IPC measures; RDM were asked to make notes of queries that were addressed

COVID, Coronavirus disease; RDM, Recently delivered mother; PPE, Personal Protection Equipment; SOP, Standard Operating Procedure; IPC, Infection prevention and control; OPD, Outpatient department; HCW, Healthcare workers.

#### Change in attitude of patients and healthcare providers with time

Toward August 2020, evidence was emerging that breastfeeding was safe during the pandemic. The COVID-19 wave in Delhi was also receding. HCPs were more confident and RDMs and family members were more receptive toward breastfeeding and rooming-in-related advice. The workload in hospitals was easing-off. The availability of PPEs had improved. Concurrently, COVID anxiety had also started waning. Provider–patient interactions improved.

#### Efficiency of counter-measures increased with time

Hospital processes had become more “COVID-adaptive” over time. Operational innovations minimized the risk of cross-infection and optimized IPC practices and the use of resources. For example, doctors scheduled clinical rounds of COVID wards after that in non-COVID wards and did the case notes documentation in the duty room instead of bedside, thus, minimizing the demand for PPEs. Staff allocation rosters were rationalized according to risk exposure and client load. Simultaneously, sensitization training and hand-holding support were also provided to the staff rotated from other specialties to the nursery and maternity wards. Crowd management practices became more efficient.

Suspension of elective services, mass movement restrictions (lock-down), and having designated COVID facilities helped in optimizing workload and improving institutional efficiency. As referral chains for transferring COVID-19 cases to designated facilities strengthened, case load on non-COVID facilities decreased. Institutional designations as exclusive COVID/non-COVID facilities made the implementation of SOPs easier, safer, and more efficient.

“Through these past 6 months, whole system has evolved so much! Initially we didn't know what to do. When we got COVID positive mother, the first question was, what to do with the baby if the baby is well. Now we have a separate step-down unit… we have COVID positive step-down nursery, we have COVID positive ward. So initially we did not have the whole setup and we used to lack on those aspects, and we have built ourselves around it.” (-Resident Doctor)

As testing capacity increased, all RDMs were tested for SARS-CoV-2. This increased confidence in the staff. The RDMs and families appreciated that the hospitals were strict with norms of social distancing and IPC and had made testing mandatory for all patients seeking admission. The RDMs, newborns, and HCPs were also monitored for COVID-19 symptoms. Symptomatic HCPs were tested and isolated, if positive. Hospitals had arranged separate accommodation, travel, and quarantine facilities for the staff on COVID-19 duty. This improved staff availability while minimizing the exposure of their families to SARS-CoV-2.

#### Making services family-centered

With time, the staff had adopted effective ways to counsel clients. HCPs had increased their availability and access for RDMs and family members. Departments now laid greater emphasis on counseling for skin-to-skin contact, breastfeeding, and rooming-in during pre-labor, labor, and at discharge to the RDMs and families. Providing detailed information on breastfeeding and newborn care as well as engagement of family members in the decision-making process led to better compliance with breastfeeding practices and overall satisfaction among the clients. Regular telephonic or video call–based updates about RDMs and their babies to the family members were initiated in two study hospitals. Reports were also shared with the family through WhatsApp messages. Several RDMs reported receiving prompt and timely treatment. The staff was also reported to be more patient, caring, and addressing their queries. Even the capacity of the support staff (e.g., guards and workers) for facilitating patients improved with experience.

“We get to talk to the doctor every day and get updates on the patient…they gave their number and they called twice in a day too! Once in the morning and once in the evening. They made two video clips and sent it to us, so that we can see the baby” (-Family member of a COVID-positive RDM)

“People say bad things about Government hospitals but my experience was very good. The doctors patiently heard and advised every patient, and we could ask them anything and they would always provide a solution for that.” (-Family member of a COVID-positive RDM)

#### Optimizing and coordinating discharge for mother–newborn dyads

Over the months, most facilities realized that if the RDM and her newborn were discharged on different days, it impeded breastfeeding. Consequently, they made efforts to align their discharge dates, i.e., only after both had completed mandatory days of institutional isolation or had tested negative for SARS-CoV-2. If rooming-in was possible, they were discharged after establishing breastfeeding.

#### Support to the RDM during hospital stay and during follow-up

One of the study hospitals had started allowing one family member per RDM to visit her in-hospital but with PPE on and within strict visiting hours. A few institutions had allowed family members into the nursery and involved them in the care of the newborn. This had improved care as well as feeding of expressed breastmilk.

As routine health services were impaired in the community, the institutions ensured that vaccination of the newborn was up to date and that the RDMs and family members had been adequately counseled for exclusive breastfeeding and after-care at home, at the time of discharge. They prioritized follow-up calls to RDM–newborn dyads that were in the high-risk category.

The use of video-calling services was a major technological innovation that one of the institutions had adopted. With funding from the Government, the institution had received hand-held electronic tablets with an internet connection—these were used by the staff in the nursery to interact with the RDMs under isolation. The RDMs in the said institution hailed this as a major enabler that allayed their anxiety as they could interact with the nursery staff as well as watch their babies being cared for in the nursery.

#### Infrastructural modifications and “zoning”

Gradually, the hospitals were able to install exhaust fans, cameras, and devices for remote monitoring and counseling of patients. Separate passages, lifts, and “red-green-yellow” zoning were done based on infection status for positive, negative, and suspected cases, respectively. Donning and doffing areas were separately ear-marked. Equipment was also procured for each zone to ensure IPC. Separate labor rooms and neonatal intensive care units (NICUs) were accordingly zoned and equipped expeditiously.

#### Guidelines coming up after initial months and their sharing and adherence by staff

The Federation of Obstetric and Gynecological Societies of India (FOGSI), National Neonatology Forum of India (NNF), and Indian Academy of Pediatrics (IAP) had come up with a joint statement titled “clinical practice guidelines for perinatal-neonatal management of COVID-19 infection” on 26^th^ March 2020 (38); an updated second version was released on 7^th^ May 2020 (39). Simultaneously, the Indian Council of Medical Research (ICMR) also actively updated its advisories. Availability and wider dissemination of these guidelines facilitated the adoption and standardization of practices by providers who were now more confident in taking decisions and counseling the patients and their family members. Departmental standard operating protocols (SOPs) were revised in view of the guidelines after internal and interdepartmental meetings. Sharing literature on social media groups, e.g., closed WhatsApp groups, webinars, and training (physically provided in small groups or through virtual platforms) improved staff confidence for coordinated care.

#### Interdepartmental coordination

The pandemic demanded efficient coordination and active engagement between Obstetric, Neonatology/Pediatric departments for optimal care of RDM–newborn dyads. As structured management protocols were evolving, coordination between these departments became dynamically adaptive. We noted that the departments that already had a smooth consensus-driven work culture could tide over the coordination challenges effectively. Day-to-day coordination was often through non-formal (not written down) understanding and accomplished over WhatsApp groups.

“…there are some coordinators in OBG team and neonatal team. Initiating breastfeeding is more of a joint effort.” (-HOD, Neonatology)

#### Leadership in promoting breastfeeding in newborns

Departmental leadership played a very critical role as an enabler (“leadership in crisis”). Active engagement in surge preparedness for IPC and pandemic combat (“leading from front”) was deemed as a major leadership trait by the participants. These “leaders” advocated for the sustenance of pre-existing quality of maternal and newborn care practices even in the COVID wards, had active involvement in updating and contextual adaptation of the SOPs, and were at the forefront of patient care and administration. They kept abreast with the fast-evolving evidence and this instilled confidence among peers and staff. Alongside, they organized frequent sessions of counseling with staff and patients and motivated them constantly, exercised high levels of emotional intelligence, and demonstrated empathy for the overworked and often anxious staff and distressed patients.

“…our practices in terms of ensuring that the baby gets 1st drop of milk and every drop of her own mother's milk, is something that we ensure from the point that the lady is in the labor room…We have (a practice that) for the mother and her baby, breastfeeding has to be established… So, we ensure it happens here.” (-HOD, Neonatology).

### Analytic framework depicting the pathways of impaction of optimal breastfeeding practices in institutionalized RDM-newborn dyads during the COVID-19 pandemic

**Figure 1:**
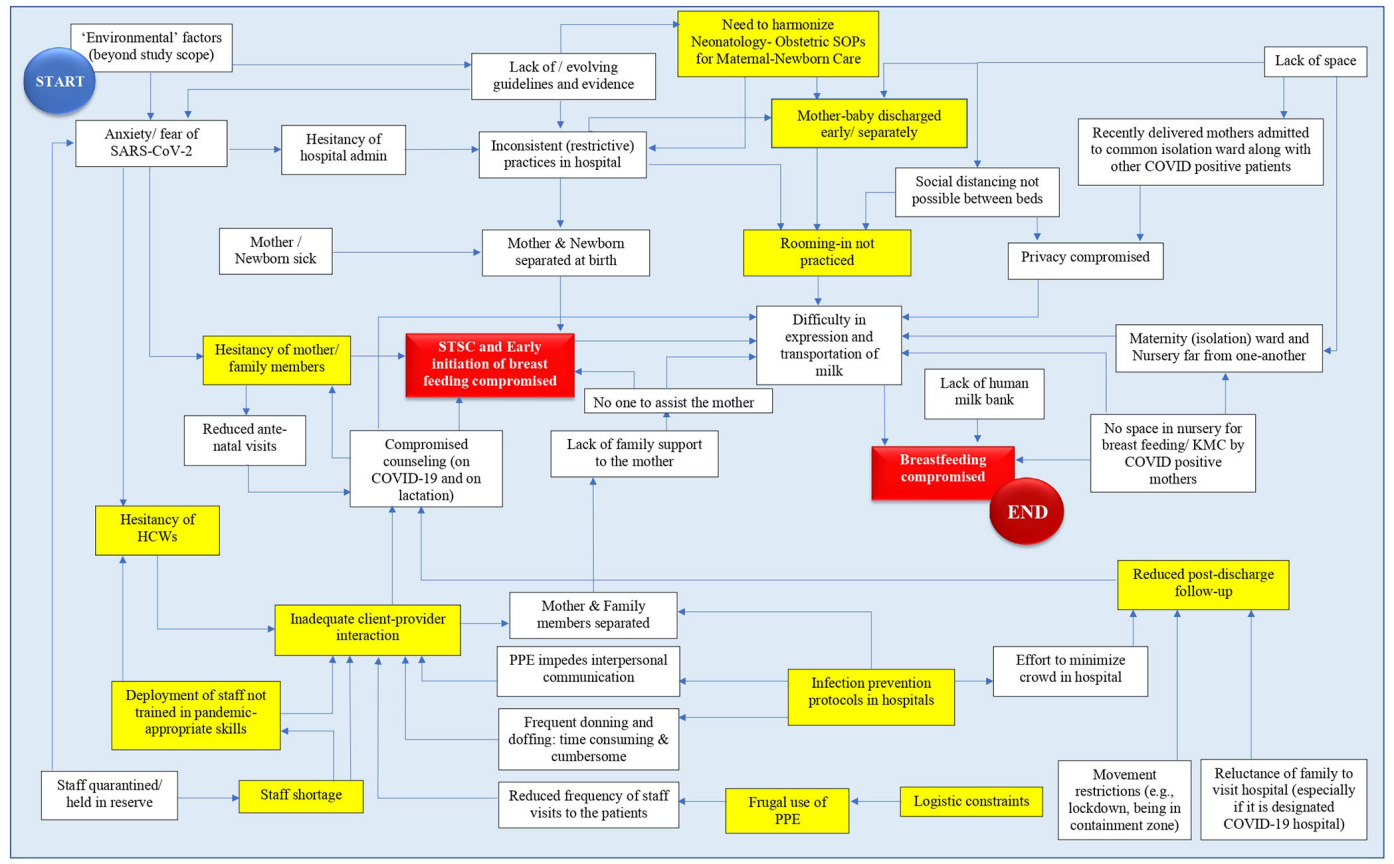
Analytic framework depicting the pathways of impaction of optimal breastfeeding practices in institutionalized mother-newborn dyads during the COVID-19 pandemic. The various barriers are depicted in the white boxes – the ones targeted by the study institution for mitigation have been highlighted in yellow. Boxes highlighted in red represent end-points; *HCW, Health Care Worker; KMC, Kangaroo Mother Care; PPE, Personal Protection Equipment; STSC, Skin-To-Skin Contact; SARS-CoV-2, severe acute respiratory syndrome coronavirus 2; COVID, Coronavirus disease; SOP, Standard Operating Procedure*.

Figure 1 shows the factors (in white and yellow boxes) influencing breastfeeding practices and their interactions (arrow heads) in institutionalized RDM–newborn dyads during the COVID-19 pandemic. The red colored boxes suggest the endpoints. Various barriers are depicted in the white and yellow boxes. The barriers highlighted in yellow are those that the institutions could intervene and overcome, at least partially.

Table 5 summarizes the suggestions for improvement coming from all participants of the study.

**Table 5:** Suggestions for improvement from the participants.

**Neonatologists/pediatricians**		
**Need for comprehensive guidelines**	**85**	**Most**
Update and adapt practices quickly according to evolving guidelines	46	Some
Design guidelines that are feasible for ground-level implementation	46	Some
All stakeholders and subject experts should come together for formulation	15	Few
Clarity of guidelines	23	Few
Need for protocols on attendants	8	Few
**Better logistics and staff management required**	**54**	**Many**
Need for more dedicated staff	38	Some
Logistic supply chain needs to be strengthened	8	Few
Need for more dedicated space	15	Few
Make staff deployment more efficient	15	Few
**Facilitate breastfeeding and newborn practices**	**54**	**Many**
Create dedicated lactation teams	46	Some
Arrange for allowing birth companions to help mothers	8	Few
Create mechanisms to reinforce exclusive breastfeeding for 6 months	8	Few
Newborn care must be practiced	8	Few
**Need for effective counseling of patients**	**46**	**Some**
Ensure skilled counseling for mothers	31	Some
Improve communication with the patients and providers for better implementation	15	Few
**Training of staff**	**38**	**Some**
Training needed for waste disposal	15	Few
Staff needs training upon return to duty from posting elsewhere	8	Few
Staff training for kangaroo mother care required	8	Few
Need to train rotated staff for newborn care	8	Few
Joint training with clear role rationalization for neonatology and obstetric staff	8	Few
Training on infection control measures and on handling patients with COVID	8	Few
**Improving inter-departmental support**	**15**	**Few**
Need to improve interdepartmental coordination	15	Few
Departments need to sensitize one another about their SOPs	8	Few
**Motivating and empowering healthcare workers**	**23**	**Few**
Identifying and complementing champions	15	Few
Need for motivational endeavors for staff	23	Few
**Prioritizing and promoting BF practices in newborn**	**23**	**Few**
Prioritize health care budget	8	Few
Promotion of breastfeeding on primetime channels by professionals and ministries	8	Few
Technological innovation	8	Few
**Obstetricians**		
**Training of staff**	**33**	**Some**
Improve communication with the patients and providers for better implementation	17	Few
Training on infection control measures or how to handle COVID	17	Few
**Facilitate better BF and newborn practices**	**17**	**Few**
Newborn care must be sustained irrespective of pandemic situation	17	Few
**Need for comprehensive guidelines**	**17**	**Few**
Provide clear guidelines	17	Few
**Need for effective counseling of patients**	**17**	**Few**
Strengthen counseling skills of staff through training	17	Few
Prioritize effective counseling of RDMs	17	Few
**Residents**		
**Requirement for better logistics and increase in staff capacity**	**50**	**Many**
Increase availability of staff for better monitoring	7	Few
Allocate more space for mother and baby to room together	36	Some
Create designated team for COVID mothers for better care	7	Few
Ensure adequate availability of equipment	7	Few
**Need for effective counseling of patients**	**50**	**Many**
Start counseling and communication even before the delivery	14	Few
Work toward improving quality of counseling	36	Some
Intensify counseling for patients with COVID	14	Few
Staff training on effective counseling of mothers	14	Few
**Need for comprehensive guidelines on optimal breastfeeding practices**	**29**	**Some**
Create robust (stable) guidelines	21	Few
Need to involve ground-level staff to formulate guidelines contextually	7	Few
**Prioritizing and promoting breastfeeding practices in newborn**	**29**	**Some**
Need for availability of group support	7	Few
Allocate dedicated specialist nurse for newborn care	7	Few
Explore options for early discharge without compromising breastfeeding practices	7	Few
Provide privacy for breastfeeding	14	Few
**Improve staff orientation to guidelines**	**7**	**Few**
**Nurses**		
**Facilitate optimal breastfeeding and newborn practices**	**54**	**Many**
Infection prevention and control protocol must ‘accommodate' rooming-in	23	Few
Have an allocated room with staff for Kangaroo mother care and breastfeeding	15	Few
Discharge and let them be in-home quarantine so that they get some help	8	Few
Allow mother to see her baby being cared at least from a distance	8	Few
Have a meeting area where the mothers could meet their family and newborn	8	Few
Facilitate transportation of expressed breastmilk from mothers to newborn	8	Few
Need to focus more on practical practice of breastfeeding than documentation	8	Few
Practice early initiation of breastfeeding and rooming-in	8	Few
**Better logistics required**	**31**	**Some**
Need for a better diet as attendants or visitors are not being allowed	8	Few
Need for more manpower	8	Few
Need for personal protective equipment kit	8	Few
Need for setup of NICU adjacent to ward with a glass wall, so that mothers can see the baby anytime they want	8	Few
Need to have more staff to help us with our work	8	Few
Postnatal ward and nursery should be near each other	8	Few
**Improve staff skills**	**31**	**Some**
Need for more detailed training on counseling during COVID	8	Few
Need for routine sensitization of healthcare workers to allay fear	15	Few
Training needed for all staff irrespective of COVID duties	8	Few
**Need for effective counseling of patients**	**31**	**Some**
Need for dedicated lactation counselors for better breastfeeding	15	Few
Need for more effective counseling especially for positive mothers	15	Few
**Need for patient education for establishing breastfeeding**	**8**	**Few**
**The neonatologist should take a leading role in media to spread awareness**	**8**	**Few**
**Healthy mother and newborn**		
Counsel all first-time mothers beforehand	6	Few
Counsel on how to clean the breasts to avoid infecting the newborn	6	Few
Provide information on alternatives to breastmilk for feeding the newborn	6	Few
Identify a person exclusively for addressing all in-patient queries	6	Few
Staff should be available to resolve queries during follow up	6	Few
Provide information on infection prevention and control practices	6	Few
**Mother with COVID-19 and healthy newborn**		
Improve treatment and behavior toward infected patients	11	Few
Provide physical support to the mother (especially if a cesarean section)	11	Few
Facilitate rooming-in	5	Few
Improve crowd management in queues for testing for SARS-CoV-2	5	Few
Provide further guidance and advice on feeding expressed milk to baby	5	Few
Provide more information related to COVID-19	5	Few
**Mother with COVID-19 and sick newborn**		
Staff needs to be more empathetic	13	Few
Provide help for expressing milk (especially, if delivered by cesarean section)	13	Few
Improve the quality of food supplied in the hospital	13	Few
Provide more information on feeding baby before discharging	13	Few
Provide daily updates on the health status of the newborn	13	Few

SOP, Standard Operating Procedure; COVID, Coronavirus disease, SAVS-CoV-2, severe acute respiratory syndrome coronavirus 2.

## Discussion

We observed that the pandemic disrupted newborn care and breastfeeding practices across RDM–newborn profiles at the study institutions through complex interrelated pathways. We identified RDM–newborn separation at birth as an important barrier that institutions largely failed to overcome in the initial months of the pandemic. In the initial months of the pandemic, there was considerable confusion and stress for healthcare providers (HCPs) and breastfeeding mothers regarding the safety of breastfeeding in RDMs with SARS-CoV-2 infection. Furthermore, the high delivery case load (~40–50 deliveries/day) at the participating facilities and lack of space precluded the possibility of rooming-in of so many suspect mothers and baby dyads in isolation wards. Institutions tried to stratify the laboring mothers by the status of SARS-CoV-2 test reports. The institutions kept the RDM baby separated until the test was reported negative. Since test results were invariably delayed, this resulted in the separation of the RDM and her newborn. Once separated, rooming-in and establishing breastfeeding became challenging. For a baby who was admitted to the nursery away from the mother, transportation of expressed breastmilk from the isolation ward to the nursery was another major barrier in our study. As a mother's visitation to the nursery to be involved in the care of her baby was limited for the fear of spreading COVID-19, there was considerable anxiety and stress among mothers that was counterproductive for their uninterrupted milk supply. We also noted that formula feeding and early discharge of the baby with alternative caregivers while the mother was still admitted in the COVID-ward became common practices.

Findings similar to ours have been reported from other countries too. In a global online survey involving 62 LMICs to provide insights on disruptions to coverage and quality of small and sick newborn care, it was found that preparedness for COVID-19 was suboptimal in terms of guidelines and availability of personal protective equipment and that the guidelines for testing of mother and newborn changed frequently. More than 85% of health personnel feared for their own health and 89% had increased stress (21). Newborn care practices were disrupted both due to reduced care-seeking and compromised and inexperienced staffing in the COVID wards. More than half reported that standard practices for small neonates such as kangaroo mother care were either discontinued or discouraged. Separation of the mother–baby dyad was reported for both COVID-positive mothers (50%) and those with unknown status (16%). Follow-up care was disrupted primarily due to families' fear of visiting hospitals (~73%). COVID-19 has also been reported to have also compromised the quality of counseling and the extent of social support to RDMs; we have also observed the same in this study (40).

Despite clear and comprehensive guidance from the WHO regarding exclusive breastfeeding, during the beginning of the pandemic (14), newborns were often separated from the mother at birth. Even professional associations, e.g., in the Philippines, Thailand, Malaysia, and the United States of America (USA) released guidelines supporting the separation (41). The separation affected breastfeeding (41–43). Some places allowed only screened donated breastmilk from COVID-19-negative mothers (44, 45). The disharmony between guidelines from the professional associations and that from the WHO was evident in the high-income countries as well. In a study (46) comparing the initial recommendations of the professional obstetrical and gynecological societies of five high-income countries, namely Australia, New Zealand (NZ), Canada, the United Kingdom (UK), and the USA, with that from the WHO (14, 16, 47), none of these aligned perfectly. Discordant guidelines impeded confident decision-making and the families received conflicting information. First-time mothers who were COVID-19 negative also reflected fear, anxiety, and doubts and often made a personal choice of not wanting to breastfeed their newborn for fear of unknowingly transmitting COVID-19 infection (test results were at times considered to be unreliable) (48). Inadequate provider–patient interaction both during hospital stay and during follow-up was another barrier to successful breastfeeding, during the COVID-19 pandemic. Nevertheless, mitigatory efforts were also responsive to the evolving evidence on the low risk of transmission of the virus through breastfeeding (49–55). For example, in April 2020, the American Association of Pediatrics recommended separating infected mothers from newborns but in July 2020, it retracted the advice (56, 57).

In the present study, the use of video-calling services was a major technological innovation that one of the institutions had adopted. This innovation was found to be a major enabler that allayed the anxiety of the mothers. Family-centered care (FCC) in the NICU focuses on building trust, reducing anxiety, partnering, and integrating families into the care of their infants. Many newborns in NICU have critical health issues causing anxiety and feelings of fear to parents and families. Separation, especially in life-threatening conditions, causes worry and apprehension among families. Providing a means for families to view live videos of their infants, any time of the day or night, and while away from the unit, can help alleviate this stress. Virtual visitation of caregivers has been used in a few NICUs in the USA and UK even before the COVID-19 pandemic (58–61). The NICVIEW is a web-based camera system (WBCS) that streams real-time video of the baby around the clock. In a questionnaire survey conducted in a Canadian hospital, following the implementation of NICVIEW for virtual visitation of caregivers during the COVID-19 pandemic due to the strict implementation of the policy of restricted entries found that 98.0% of parents felt very connected with their baby, and almost 90.0% of parents reported having the WBCS helped reduce their anxiety and stress levels to a great extent. An accepting attitude from HCPs, especially the nursing staff, is of paramount importance in improving the successful implementation of WBCS in the NICU.

While family centricity is important, HCP centricity was also critical during the early days of the pandemic, since it kept the HCPs confident and motivated. In a large hospital-based prospective cohort study from India with high patient load and resource limitations, a dynamic policy using a low-cost paperless communication system with mobile devices was reported to reduce the risk of HCP infection, improve their motivation, and re-deployment in the COVID wards (62). There are reports that leadership skills were useful during the pandemic for delivering essential facilities and services rapidly and with empathy during the pandemic (63–65). In the present study, we recorded leadership-in-crisis skills as helpful in mounting up a coherent response within the healthcare institutions, e.g., in motivating the staff, by rapidly delegating authority to organizational management, framing and updating standard operation procedures as per changing guidelines for the management of pregnant women and their neonates with suspected or proven COVID-19 infection, allaying anxiety of patients and providers, by innovating for improving neonatal and follow up care, and interdepartmental coordination, besides facilitating the mobilization of resources. This guiding coalition had an overriding vision to contain the infectious disease throughout the pandemic, while simultaneously catering to patients with and without COVID-19 infection. In the present study, other enablers which played a critical role in improving neonatal care and breastfeeding rates have conducive attitudes and neonatal care practices with the passage of time. Certain systemic interventions were also helpful e.g., designating some facilities exclusively for COVID-19 care, strengthening referral pathways, and release of joint statements by FOGSI-NNF-IAP. FOGSI-NNF-IAP Joint Statement greatly helped practitioners take confident decisions related to sustaining routine pre-pandemic practices for breastfeeding and newborn care (29). We infer that in case of a public health crisis, national health agencies and professional associations must ensure that coherent guidelines are released at the earliest and disseminated widely along with strategies to counter misinformation.

### Strengths and limitations

We pooled information from clients and providers across seven premier institutions in Delhi, which had pre-existing systems for evidence-based optimal newborn care practices. This gave us an opportunity to identify deviations from the “near-ideal.” However, we acknowledge that each institution had its own unique strengths and weaknesses, and our aggregated analysis does not delve deeper into these. To address the influence of temporal change, we requested the participants to respond with the initial 6 months of the pandemic (March-August 2020) as the reference period. We could not explore post-discharge breastfeeding practices.

## Conclusion

We recommend against separating the mother and the newborn during public health emergencies without robust evidence favoring the same. We call for making routine RDM–newborn care practices family-centered at all times. We encourage the practice of early release of joint statements by professional associations and authorities during public health emergencies. Leveraging social media and video conferencing technology, having dedicated lactation teams, investing in improved hospital architecturalplanning, and having an in-campus human milk bank could provide an advantage against potential disruption and merit further research.

The learning of this study should help in building institution-based newborn care services more resilient against disruptions by public health emergencies.

## Data availability statement

The raw data supporting the conclusions of this article will be made available by the authors, without undue reservation.

## Ethics statement

The Institutional Ethics Committee of ABVIMS and Dr. Ram Manohar Lohia Hospital, New Delhi reviewed and approved the study protocol. Administrative approval was provided by the Ministry of Health and Family Welfare. Written informed consent for participation was not required for this study in accordance with the national legislation and the institutional requirements.

## Author contributions

AMa: concepts, design, definition of intellectual content, tool development, manuscript editing, manuscript review, and guarantor. RM: tool development, literature search, data acquisition, data analysis, manuscript preparation, and manuscript editing. SU, KPr, and TB: data acquisition, manuscript editing, and manuscript review. RG, BD, AS, PM, MS, and KPa: manuscript review. PP: tool development, literature search, data acquisition, and manuscript review. AMo: concepts, design, definition of intellectual content, tool development, data acquisition, data analysis, manuscript preparation, manuscript editing, and guarantor. All authors contributed to the article and approved the submitted version.
