# Supplementary material for: Barriers and enablers of breastfeeding in mother–newborn dyads in institutional settings during the COVID-19 pandemic: A qualitative study across seven government hospitals of Delhi, India
Source: Front Nutr. 2022 Dec 8;9:1052340. doi: 10.3389/fnut.2022.1052340 (PMC9773092; doi:10.3389/fnut.2022.1052340)
Supplement: Supplementary file 1 [file Data_Sheet_1.docx]

**S1: Supplementary Box and Table**

| **Supplementary Box 1\|** |
| --- |
| **Study instruments:**   1. **Development: ‘**Determinants of breastfeeding’ is a fairly well-researched topic in India. However, the COVID-19 situation is unprecedented. Much adaptation and deviation from routine practice in this regard has happened ever since the pandemic showed up in India. To start with, we reviewed relevant literature and guidance documents from the World Health Organization (WHO), Ministry of Health and Family Welfare (MOHFW) - Government of India (GOI), Indian Council of Medical Research (ICMR), Breastfeeding Promotion Network of India (BPNI), Indian Academy of Pediatrics (IAP), and Federation of Obstetric and Gynaecological Societies of India (FOGSI). We organized a group discussion with the following team members - Unit head (Neonatology, RMLH), four faculty members (Neonatology, RMLH), lactation counsellor (RMLH), resident (Department of Obstetrics), and two invited extramural researchers (1 community medicine expert, 1 psychologist). The group discussed the evidence and guidance from available literature and prepared a careseeking pathway for a pregnant woman presenting to the hospital during the pandemic, for delivery, till her and her newborn’s discharge from the hospital. The team highlighted the potential determinants (barriers and enablers) at each step from lived experience and also what potential questions were of utmost priority for exploration at each of these. Subsequently, each participant was requested to independently submit at least five items of enquiry for each participant category. A team of qualitative researchers then consolidated the items into a draft interview schedule for each participant category. 2. **Validation:** Once the draft instruments were ready, these were reviewed by the team for construct, content and face validity independently and in group. Items that were redundant were removed, queries were added/ edited for more comprehensive exploration of constructs, and grammar and sequence was refined for face validity and flow of the interviews. The items were prepared in English and then translated to Hindi and then back translated to English for accuracy. Following an orientation session about standardized interviewing techniques, the team administered each tool to two candidates whose profile matched that with potential participants, in Hindi/ English as per their preference (pretest 1). During the administration, the participants were asked to point-out any item that they could not understand or were uncomfortable to answer. The interviewers also took a note of any need for simplifying the language or rearranging the sequence of the items in the tools. The team then revised the tools as appropriate, in consensus, and administered them to another set of two participants (pre-test 2) before finalization. 3. **Piloting:** The tools were piloted on the participants in each category at one of the study sites. The piloting exercise was majorly for examining the operational feasibility of the study. It was observed that participants were more forthcoming if they were interviewed with prior appointment. Subsequently, the team ensured that the participants were informed about the interview and the interviewers at least a day in advance and an appointment for about an hour secured for the next day. No changes had to be made to the tools during or after the piloting. 4. **Administration:** The interviewers were trained about qualitative research methods, interviewing techniques and data recording procedures over a half-day workshop by two researchers (AM and RM) with about 10 years of experience in qualitative research methods and their application in public health research. The trainings included about eight hours of discourse on qualitative research methods and interviewing techniques. The training included didactic lectures using Power-point slides (about 2 hours) and six hours of interactive question and answer sessions. This was followed by demonstration of about five in-depth interviews and non-formal interactions each, and then conduct of at least one interview per trainee under supervision of the trainer (AMo/ RM). Subsequently, the interviewers conducted the remaining interviews in pairs at the hospital/ office of the participant, or over virtual meetings (as preferred by the participant). The questions were administered verbatim by the interviewers to maintain standardization. |

| **Supplementary Table 1\|** Profile of recently delivered mothers who participated in the study (N=45) | | | | | | |
| --- | --- | --- | --- | --- | --- | --- |
| **Variable** | **n** | **%** |  | **Variable** | **n** | **%** |
| **Religion** |  |  |  | **Rooming-in with mother** | | |
| Hindu | 37 | 82.2 |  | Yes | 25 | 55.6 |
| Muslim | 07 | 15.6 |  | No | 20 | 44.4 |
| Others | 01 | 2.2 |  | **Total** | **45** | **100.0** |
| **Total** | **45** | **100.0** |  |  |  |  |
|  |  |  |  | **Type of feed** |  |  |
| **Caste** |  |  |  | Breastmilk | 25 | 55.6 |
| Scheduled castes/ tribes | 05 | 11.1 |  | Only top feeding | 07 | 15.6 |
| Other backward classes | 08 | 17.8 |  | Mixed feeding | 13 | 28.9 |
| Others | 32 | 71.1 |  | **Total** | **45** | **100.0** |
| **Total** | **45** | **100.0** |  |  |  |  |
|  |  |  |  | **Mode of feeding** |  |  |
| **Education (No. of years completed)** | | |  | Breastfed | 20 | 44.4 |
| 0-5 years | 07 | 15.6 |  | Expressed breastmilk (EBM) | 05 | 11.1 |
| 6-10 years | 05 | 11.1 |  | Top feeding | 06 | 13.3 |
| 11-12 years | 09 | 20.0 |  | EBM + Top feed | 09 | 20.0 |
| 13-15 years | 06 | 13.3 |  | Breastfed + Top fed | 05 | 11.1 |
| >15 years | 18 | 40.0 |  | **Total** | **45** | **100.0** |
| **Total** | **45** | **100.0** |  |  |  |  |
|  |  |  |  |  |  |  |
| **Parity** | | |  | **Any pre lacteal feed given** | | |
| Primipara | 20 | 44.4 |  | Yes | 20 | 44.4 |
| Multipara | 25 | 55.6 |  | No | 22 | 48.9 |
| **Total** | **45** | **100.0** |  | Don't know | 03 | 06.7 |
|  |  |  |  | **Total** | **45** | **100.0** |
| **Socioeconomic status** | | |  |  |  |  |
| Economically Weaker Section (govt. certified) | 04 | 8.9 |  | **Baby offered skin-to-skin contact** | | |
| Others | 41 | 91.1 |  | Yes | 17 | 37.8 |
| **Total** | **45** | **100.0** |  | No | 28 | 62.2 |
|  |  |  |  | **Total** | **45** | **100.0** |
|  |  |  |  |  |  |  |
| **Type of family** | | |  | **Breastfeeding initiated within 1 hour of delivery** | | |
| Nuclear | 10 | 22.2 |  | Yes | 22 | 48.9 |
| Three Generational | 05 | 11.1 |  | No | 23 | 51.1 |
| Joint | 30 | 66.7 |  | **Total** | **45** | **100.0** |
| **Total** | **45** | **100.0** |  |  |  |  |
|  |  |  |  |  |  |  |
| **Residence** | | |  | **Baby fed colostrum** | | |
| Rural | 6 | 13.3 |  | Yes | 36 | 80.0 |
| Urban (non-slum) | 32 | 71.1 |  | No | 09 | 20.0 |
| Urban (slum) | 07 | 15.6 |  | **Total** | **45** | **100.0** |
| **Total** | **45** | **100.0** |  |  |  |  |
|  |  |  |  | **Mother feed baby multiple times on day 1** | | |
| **Mode of Delivery** | | |  | Yes | 26 | 57.8 |
| Normal vaginal delivery | 22 | 48.9 |  | No | 19 | 42.2 |
| Cesarean section | 23 | 51.1 |  | **Total** | **45** | **100.0** |
| **Total** | **45** | **100.0** |  |  |  |  |
|  |  |  |  | **Baby roomed in with mother on day 1** | | |
| **Sex of the newborn** | | |  | Yes | 26 | 57.8 |
| Boy | 25 | 55.6 |  | No | 19 | 42.2 |
| Girl | 20 | 44.4 |  | **Total** | **45** | **100.0** |
| **Total** | **45** | **100.0** |  |  |  |  |
|  |  |  |  | **Mother counselled on breastfeeding on day 1 by hospital staff** | | |
| **Pregnancy type** | | |  | Yes | 23 | 51.1 |
| Single | 45 | 100.0 |  | No | 22 | 48.9 |
| Multiple | 0 | 0.0 |  | **Total** | **45** | **100.0** |
| **Total** | **45** | **100.0** |  |  |  |  |
|  |  |  |  |  |  |  |
| **Sick newborn in NICU** | | |  |  |  |  |
| Yes | 08 | 17.7 |  |  |  |  |
| No | 3337 | 82.3 |  |  |  |  |
| **Total** | **45** | **100.0** |  |  |  |  |

**S2: Study tools (Interview guides*)***

**Unique ID------------------**

**Barriers and enablers to optimal breastfeeding practices in institutionalized recently delivered mothers during the COVID-19 pandemic: An exploratory study from New Delhi, India**

**SCHEDULE FOR UNIT HEAD**

**(Neonatology/ Obstetrics)**

1. **INTERVIEW DETAILS**

| - 1. **Site** | RML LNJP GTB AIIMS SJ  KSCH |
| --- | --- |
| - 1. **Place of interview** | Maternity ward COVID ward Other (specify) |
| - 1. **Date of interview (DD-MM-YY)** |  |
| - 1. **Start time (HH:MM)** |  |
| - 1. **End time (HH:MM)** |  |
| - 1. **Interviewer name** |  |
| - 1. **Interviewer signature** |  |

1. **PARTICIPANT PROFILE**

| - 1. **Name** |  |
| --- | --- |
| - 1. **Age (in completed years)** |  |
| - 1. **Gender** |  |
| - 1. **Designation** |  |
| - 1. **Duration of employment (in this institute)** |  |

**3. SCHEDULE FOR IN-DEPTH INTERVIEW (IDI)**

**3.1 Key instructions for the interviewer before starting the IDI**

Dear Interviewer,

- Please introduce each question verbatim, separately and record the response.
- If the respondent is unable to understand the question, repeat verbatim at a slower pace. If still unable to understand, ask which word is difficult to understand so that you could explain that in your own language.
- Please probe as suggested but do not prompt
- Please record the proceedings using the tape recorder/digital recorder with due labelling
- Please also record the verbatim responses in blank sheets provided for the question
- The elicited information is confidential

**3.2 Introductory statement (To be read out aloud verbatim to the participant)**

*Namaskar!*

*Thank you for consenting to participate in this interaction. I am ____________________ from the Department of Neonatology, Ram Manohar Lohia Hospital, New Delhi. I am accompanied by ______________. This interaction will last for about 45 minutes. During this while, I will ask you some questions by reading out from a document. Please respond to these to the best of your capacity and interest. We will keep all the information confidential and you will not be identified in any of the project results. The information you share will be used only for research purpose in order to improve maternal and newborn care. Your facilitation and support is critical in this and we seek your valuable inputs for understanding the issues.*

1. **Questions (to be administered verbatim)**
   1. How has the COVID-19 pandemic affected breastfeeding promotive approaches in your unit? (Probe: skin to skin care, initiation of breastfeeding, rooming-in/ bedding-in, feeding of the newborn, allowing birth attendant)
   2. What modifications did you have to make in order to accommodate infection prevention and control protocols in your day-to-day care of the newborn and its feeding? (Probe: evolution of practices since the beginning of COVID-19, separate COVID wards for RDMs, infrastructure, logistics)
   3. Are you testing all mothers for COVID-19? Can you briefly describe your practices around it?
   4. What challenges do you face in implementing the existing guidelines for breastfeeding? How do you mitigate these?
   5. What are the best practices in your unit for promoting optimal breastfeeding in recently delivered mothers? (Probe: for COVID-19 status)
   6. How do you reinforce optimal breastfeeding practices among recently delivered mothers once they are discharged? (Probe: during inpatient care, pre-discharge counselling, follow-up)
   7. How do you think COVID-19 related anxiety and stigma has impacted practices regarding care of RDM-newborn dyads in your unit/ department? (Probe: role of authorities, staff and family members)
   8. What areas of work, according to you needs further skill building for your staff? How do you think this could be best addressed?
   9. What challenges are you facing in terms of staff allocation for COVID duty? How do you think this has impacted practices in your unit/ department? (Probe: deployment of senior staff during labour and in post-natal ward, staff reluctance / absenteeism, 24x7 staff availability)
   10. How do you mitigate staff shortage? (Probe: rotation/ deputation of staff from other departments; if yes, ask about level of seniority, training and proficiency)
   11. How do you ensure inter-departmental (obstetrics-neonatology) coordination while managing RDM-newborn dyads? (Probe: areas needing special attention)
   12. What policy and practice level preparedness would you recommend for ensuring optimal breastfeeding during future influenza pandemics?
   13. Anything you would like to add?

**Thank you!**

**Unique ID-----------------**

**Barriers and enablers to optimal breastfeeding practices in institutionalized recently delivered mothers during the COVID-19 pandemic: An exploratory study from New Delhi, India**

SCHEDULE FOR RESIDENTS

| - 1. **Site** | RML LNJP GTB AIIMS SJ KSCH |
| --- | --- |
| - 1. **Place** | Maternity ward COVID ward Other (specify) |
| - 1. **Date (DD-MM-YY)** |  |
| - 1. **Start time (HH:MM)** |  |
| - 1. **End time (HH:MM)** |  |
| - 1. **Interviewer name** |  |
| - 1. **Interviewer signature** |  |

1. **INTERVIEW DETAILS**

**2. PARTICIPANT PROFILE**

| - 1. **Name** |  |
| --- | --- |
| - 1. **Age (in completed years)** |  |
| - 1. **Gender** |  |
| - 1. **Designation** |  |

**3. SCHEDULE FOR IN-DEPTH INTERVIEW (IDI)**

**3.1 Key instructions for the interviewer before starting the IDI**

Dear Interviewer,

- Please introduce each question verbatim, separately and record the response.
- If the respondent is unable to understand the question, repeat verbatim at a slower pace. If still unable to understand, ask which word is difficult to understand so that you could explain that in your own language.
- Please probe as suggested but do not prompt
- Please record the proceedings using the tape recorder/ digital recorder with due labelling
- Please also record the verbatim responses in blank sheets provided for the question
- The elicited information is confidential

**3.2 Introductory statement (To be read out aloud verbatim to the participant)**

*Namaskar!*

*Thank you for consenting to participate in this interaction. I am ____________________ from the Department of Neonatology, Ram Manohar Lohia Hospital, New Delhi. I am accompanied by ______________. This interaction will last for about 45 minutes. During this while, I will ask you some questions by reading out from a document. Please respond to these to the best of your capacity and interest. We will keep all the information confidential and you will not be identified in any of the project results. The information you share will be used only for research purpose in order to improve maternal and newborn care. Your facilitation and support is critical in this and we seek your valuable inputs for understanding the issues.*

1. **Questions (to be administered verbatim)**
   1. How have you adapted your practices for early newborn care in view of the COVID-19 situation? (Probe: if it has been missed out:
      - Role of birth companion
      - Skin-to-skin care & initiation of breastfeeding
      - rooming-in/ bedding-in
   2. As regards optimal breastfeeding among recently delivered mothers, how have your practices changed ever since the COVID-19 pandemic has set in? Why? (Probe:
      - workload, PPE usage
      - infrastructure issues, isolation needs
      - quality of interaction with the patient
   3. What according to you are the most critical barriers to breastfeeding in the hospital during the pandemic? How do these impact counselling strategies? (Probe:
      - method, content and frequency of counselling
      - receptivity of the recently delivered mother and family to the counselling
      - feeding of colostrum & exclusive breastfeeding
   4. In view of the COVID-19 situation, you may be advising for expression and feeding of breastmilk in some mother-newborn dyads. Could you please describe what challenges you have experienced in doing so and how you managed these? (Probe:
      - staff capacity
      - breast pumps, wet-nursing, human milk bank (if applicable)
      - transportation and feeding of milk to the baby
   5. According to you, what are the major challenges to decision making with regard to breastfeeding practices in recently delivered mothers in your institution? why do you say so? (Probe:
      - COVID-19 guidelines and training needs
      - ethics, consent and documentation related challenges
      - discharge and follow-up practices
   6. Do you think you could have done better for ensuring optimal breastfeeding practices in the recently delivered mothers? Why do you say so?
   7. Anything that you would like to add?

**Thank you!**

**Unique ID-----------------**

**Barriers and enablers to optimal breastfeeding practices in institutionalized recently delivered mothers during the COVID-19 pandemic: An exploratory study from New Delhi, India**

SCHEDULE FOR LACTATION COUNSELLOR/ DEDICATED STAFF NURSE FOR BREASTFEEDING COUNSELLING (Hindi)/ DELIVERY ROOM STAFF NURSE

**INTERVIEW DETAILS**

साक्षातकार [विवरण](https://www.shabdkosh.com/dictionary/hindi-english/%E0%A4%B5%E0%A4%BF%E0%A4%B5%E0%A4%B0%E0%A4%A3/%E0%A4%B5%E0%A4%BF%E0%A4%B5%E0%A4%B0%E0%A4%A3-meaning-in-english)

| - 1. **Site**   स्थान | RML LNJP GTB AIIMS SJ KSCH |
| --- | --- |
| - 1. **Place of interview**   साक्षातकार की जगह | Maternity ward मेटरनीटी वार्ड  COVID ward कोविड वार्ड  Other (specify) अन्य |
| - 1. **Date of interview (DD-MM-YY)**   साक्षातकार की तारीख (DD-MM-YY) |  |
| - 1. **Start time**   शुरुआत का समय | (HH: MM) |
| - 1. **End time**   समाप्त करने का समय | (HH: MM) |
| - 1. **Interviewer name**   साक्षातकार लेने वाले का नाम |  |
| - 1. **Interviewer signature**   साक्षातकार लेने वाले का हस्ताक्षर |  |

1. **Interview details**:
2. **PARTICIPANT PROFILE (Triangulated from interaction, direct observation and hospital records)**

प्रतिभागी की जानकारी

| - 1. **Name**   नाम |  |
| --- | --- |
| - 1. **Age (in completed years)**   उम्र (पूर्ण वर्षों में) |  |
| - 1. **Gender**   लिंग |  |
| - 1. **Designation**   पद |  |
| - 1. **Duration of employment in this hospital**   इस अस्पताल में नौकरी की अवधि |  |
| - 1. **Academic qualification**   शैक्षणिक योग्यता |  |

**3.** साक्षातकार **schedule**

**3.1** Dear Interviewer,

- Please introduce each question verbatim, separately and record the response.
- If the respondent is unable to understand the question, repeat verbatim at a slower pace. If still unable to understand, ask which word is difficult to understand so that you could explain that in your own language.
- Please probe as suggested but do not prompt
- Please record the proceedings using the tape recorder/digital recorder with due labelling
- Please also record the verbatim responses in blank sheets provided for the question
- The elicited information is confidential

साक्षातकार लेने वाले के लिए अनुदेश (क़ृपया सारे सवाल मौखिक पुछे और आवाज रिकार्ड करें)।

- - अगर प्रतिवादी को कोई सवाल समझने में कठिनाई हो, तो सवाल को धीरे से पूछे और जो शब्द उसे समक्ष नहीं आ रहे है उसे विस्तार से उसकी भाषा में बताएं।
  - प्रतिवादी को स्वंय जवाब देने दे, बीच – बीच में आप उससे उचित बाते पूछे परन्तु उसे किसी जवाब के लिए प्ररेरित न करें।
  - कृपया साक्षातकार को टेप रिकार्डर या डिजिटल रिकार्डर में रिकार्ड करे, लेबल के साथ।
  - मौखिक प्रतिउत्तर को एक खाली पन्ने पर भी लिखित रूप से रिकार्ड करें।
  - बताई गई सारी जानकारी गोपनीय रखी जाएगी।

**3.2 Introductory statement (To be read out aloud verbatim to the participant)**

*Namaskar!*

*Thank you for consenting to participate in this interaction. I am ____________________ from the Department of Neonatology, Ram Manohar Lohia Hospital, New Delhi. I am accompanied by ______________. This interaction will last for about 45 minutes. During this while, I will ask you some questions by reading out from a document. Please respond to these to the best of your capacity and interest. We will keep all the information confidential and you will not be identified in any of the project results. The information you share will be used only for research purpose in order to improve maternal and newborn care. Your facilitation and support is critical in this and we seek your valuable inputs for understanding the issues.*

*नमस्कार!*

*आपका इस साक्षात्कार में शामिल होने के लिए बहुत बहुत शुक्रिया । मैं .................... हूँ। यह साक्षात्कार लगभग ४५ मिनट्स चलेगा। इस दौरान मैं आपसे कुछ सवाल पूछूँगी/ पूछूंगा, इन दस्तावेज़ को देख कर। आपसे अनुरोध है की आप अपनी क्षमता और इच्छा के अनुसार जवाब दें। हम आपकी पहचान और यह सारी जानकारी गोपनीय रखेंगे । यह जानकारियां सिर्फ माँ और बच्चे को स्वास्थय लाभ पहुँचाने के लिए उपयोग की जाएँग।*

1. **Questions (to be administered verbatim)**

पूछे जाने वाले सवाल

- 1. How has the COVID-19 pandemic affected your usual way of counselling of recently delivered mothers in your hospital for feeding of their newborns? (Probe: frequency of post-natal visits, time spent with the RDM, workload, infrastructure issues, isolation needs, PPE usage)

आप जो अस्पताल में माँ को newborn feeding की काउंसलिंग करती हो, उस पर corona pandemic का क्या असर पड़ा है?

(प्रोब:

- पोस्ट -नेटल वार्ड में बार बार जाना आना, माँ के साथ समय बिताना ,
- Workload, PPE का इस्तेमाल
- Isolation की ज़रुरत , अस्पताल के ढाँचे में बदलाव
  1. What according to you are the most critical barriers to effective lactational counselling during the pandemic? How have you addressed these? (Probe: need, method, timing, content and frequency of counselling)

आपके अनुसार इस corona pandemic के दौरान असरदार lactation counseling करने में सबसे बड़ी दिक्कतें कौन-कौनसी हैं ? आपने उसके लिए क्या उपाय किये ? (प्रोब: ज़रुरत, तरीका, किस समय, कितनी बार और क्या सलाह )

- 1. In view of the COVID-19 situation, how have you adapted your practices for newborns delivered in your hospital for COVID-positive mothers with:
  - healthy newborns
  - sick/ weak newborns?

COVID positive माओं के newborn babies की देखभाल कर पाने के लिए, आपने अपने तरीकों में क्या बदलाव किये? (Probe: स्वस्थ बच्चे ; बीमार या कमज़ोर बच्चे )?

- 1. In view of the COVID-19 situation, how have you adapted your practices for newborns delivered in your hospital for COVID- negative mothers?

इस corona pandemic के दौरान, आपने COVID Negative माओं के baby की देखभाल में क्या बदलाव किया?

- 1. In view of the COVID-19 situation, you may be advising for expression and feeding of breastmilk in some mother- newborn dyads. Could you please describe what challenges you have experienced in doing so and how have you managed these? (Probe:
     - need for patient education
     - staff capacity
     - breast pumps (procurement, use, re-use), wet-nursing, human milk bank (if applicable)
     - transportation and feeding of milk to the baby

Corona के चलते, आपने कुछ माओं को अपना दूध निकाल कर baby को पिलाने की सलाह दी होगी. क्या आप बता सकते हैं की इसमें आपको क्या दिक्कतें आयीं और आपने उसके लिए क्या किया।

[प्रोब:

- - - माँ को सिखाने की ज़रुरत
    - Staff की क्षमता
    - Breast pump (खरीदना, इस्तेमाल और दुबारा इस्तेमाल करना), दूसरी माओं द्वारा स्तनपान कराना, ह्यूमन मिल्क बैंक (जहाँ उपयुक्त हो)
    - निकाले हुए दूध को बच्चे तक पहुंचाना और उसे पिलाना
  1. How receptive have recently delivered mothers been to breastfeeding advice ever since the COVID-19 pandemic has set in? What according to you determines such reception? (Probe: at childbirth, during hospital stay, at discharge, follow-up)

Corona के चलते, जब आप स्तनपान की सलाह देती हैं, तो माँ उसे अपनाने के लिए कितनी तैयार रहती हैं और क्यों? (प्रोब: डिलीवरी के समय, अस्पताल में रहने के दौरान , छुट्टी के समय, दुबारा दिखने आएं तब)

- 1. Which strategies of yours seems to have been most effective for reinforcing optimal newborn feeding practices and why? (Probe: how have practices evolved since the beginning of COVID-19, what improves your efficiency despite the COVID-19 situation?)

माँ बच्चे को ठीक से दूध पिलाये, उसके लिए आप जो तरीके बताती हैं, उनमे से कौन- कौनसे तरीके सबसे असरदार हैं और क्यों? (प्रोब: महामारी की शुरुआत से लेकर अब तक, आपके तरीकों में कैसे कैसे और क्या बदलाव आया है ? COVID के समय कौनसी चीज़ें आपकी performance को support करती है?)

- 1. Do you think you could have done better for ensuring optimal breastfeeding practices in the recently delivered mothers? Why do you say so? (Probe: COVID-19 guidelines and training, documentation, follow-up, overall client satisfaction)

Breastfeeding se related वो कौनसी चीज़ें हैं जो आपको लगता है की आप और बेहतर कर सकती थी? (प्रोब: COVID 19 की guidelines और training, रिकॉर्ड, mothers की overall satisfaction)

- 1. Anything that you would like to add?

आप कुछ और बताना चाहेंगी?

**Thank you!**

धन्यवाद!

**Unique ID-------------**

**Barriers and enablers to optimal breastfeeding practices in institutionalized recently delivered mothers during the COVID-19 Pandemic: An Exploratory Study form New Delhi, India**

**Schedule for Recently Delivered Mothers**

| - 1. **Site**   स्थान | RML LNJP GTB AIIMS SJ KSCH |
| --- | --- |
| - 1. **Place of interview**   साक्षातकार की जगह | Maternity ward मेटरनीटी वार्ड  COVID ward कोविड वार्ड  Other (specify) अन्य |
| - 1. **Date of interview (DD-MM-YY)**   **साक्षात्कार की तारीख (DD-MM-YY)** |  |
| - 1. **Start time**   **शुरुआत का समय** | (HH: MM) |
| - 1. **End time**   **समाप्त करने का समय** | (HH: MM) |
| - 1. **Interviewer name**   साक्षातकार लेने वाले का नाम |  |
| - 1. **Interviewer signature**   साक्षातकार लेने वाले का हस्ताक्षर |  |

1. **INTERVIEW DETAILS**

साक्षातकार [विवरण](https://www.shabdkosh.com/dictionary/hindi-english/%E0%A4%B5%E0%A4%BF%E0%A4%B5%E0%A4%B0%E0%A4%A3/%E0%A4%B5%E0%A4%BF%E0%A4%B5%E0%A4%B0%E0%A4%A3-meaning-in-english)

1. **PARTICIPANT PROFILE (Triangulated from interaction, direct observation and hospital records)**

**प्रतिभागी की जानकारी**

| - 1. **Name**   नाम |  |
| --- | --- |
| - 1. **Age (in completed years)**   **उम्र (पूर्ण वर्षों में)** |  |
| - 1. **Religion**   धर्म़ | Hindu / Muslim/ Christian / Sikh / Others  हिन्दू / मुस्लिम / ईसाई / सिख / अन्य |
| - 1. **Caste**   जात | SC-ST / OBC / Others  अनुसूचित जाती- अनुसूचित जनजाति / ओ.बी.सी / अन्य |
| - 1. **Education (no. of years completed)**   **शिक्षा (पूर्ण वर्षों की संख्या)** | 0-5 / 6-10 / 11-12 / 13-15/ >15 |
| - 1. **Parity**   पेरटी | Primi / Multi  प्राइमी / मल्टी |
| - 1. **Socio-economic status**   सामजिक आर्थिक स्तर | EWS (Govt certified) / Others  ई. डब्लू. एस ( सराकार द्रारा प्रमाणित)/ अन्य |
| - 1. **Type of family**   परिवार के प्रकार | Nuclear / 3-generational / Joint  न्यूकियर / 3-जेनेरेसन / एक साथ |
| - 1. **Residence**   रहने का स्थान | Rural/ Urban (non-slum) / Urban (slum)  ग्रामीण/ शहरी/ शहरी (बस्ती) |
| - 1. **Mode of delivery**   डिलिवरी का प्रकार | NVD / CS  एऩ.वी.ट.डी. (नोरमल) / सिजेरियन |
| - 1. **Any health issue (please specify)**   **कोई और स्वास्थय समस्या** |  |
| - 1. **COVID-19 status**   कोवी़ड-19 की स्थिति | Positive / Negative / Recovered /  Not known / Choose not to disclose  पाज़िटिव/ नेगीटीव/ रिकवर्ड/ नहीं पता/ नहीं बताना चाहते। |
| - 1. **Date and time of discharge**   तारीख और समय डिस्चार्ज की |  |

1. **NEWBORN PROFILE (Triangulated from non-formal interaction, direct observation and hospital records)**

**नवजात की जानकारी**

| - 1. **Sex**   लिंग | Boy / Girl  लड़का / लड़की |
| --- | --- |
| - 1. **Date of birth (DD/ MM/ YY)**   जन्म की तारीख |  |
| - 1. **Birth weight (in kg)**   जन्म के समय वजन | <1.2 / 1.2-1.5 / 1.5-2.0 / 2.0-2.5 / 2.5-4.0 / >4.0 |
| - 1. **Gestation (number of completed weeks at delivery)**   **गर्भावस्था (पूर्ण सप्ताह की संख्या )** |  |
| - 1. **Pregnancy type**   प्रेगनेन्सी का प्रकार | Single / Multiple  सिंगल / मल्टीपल |
| - 1. **Sick requiring NICU care**   **क्या बेबी को NICU की ज़रुरत पड़ी ?** | Yes / No  हॅा / नही |
| - 1. **Any other health condition (specify)**   बच्चे में कोइ अन्य बीमारी / कमजोरी |  |
| - 1. **Is the baby currently roomed in with the mother?**   **क्या बच्चा इस समय माँ के साथ है?** | Yes / No  हॅा / नही |
| - 1. **Type of feed**   कौन सा दूध पिलाते हो | Breast milk / Only top feeding / Mixed feeding  मॅा का दूध / केवल उपर का दूध / दोनों |
| - 1. **Mode of feeding**   दूध पिलाने का तरीका | Breast fed / Expressed breast milk / Top feeding  मॅा का दूध / मॅा का दूध निकाल कर पिलाने / कोई दूसरा दूध ? |
| - 1. **Was the baby given any pre-lacteal?**   बच्चे को मॅा के दूध के अलावा भी कुछ दिया है। | Yes/ No  हॅा / नही |
| - 1. **Was the baby offered skin-to-skin care at birth?**   क्या बच्चे को जन्म के समय मॅा की छाती से तुरंत लगाया था। | Yes/ No  हॅा / नही  If yes, specify duration in minutes: ------  यदि हाँ , तोह कितनी देर तक लगाया , मिनटों में बताएं : |
| - 1. **Was the baby initiated on breast feeding within 1 hour of delivery?**   क्या बच्चे को जन्म के 1 घण्टें के अन्दर ही मॅा का दूध पिलाया था। | Yes/ No  हॅा / नहीं |
| - 1. **Was the baby fed colostrum?**   क्या बच्चे को मॅा का गाढा पीला दूध दिया गया था। | Yes/ No  हॅा / नहीं |
| - 1. **Did the mother feed multiple times on Day 1?**   क्या बच्चे को पहले दिन मॅा का दूध बार –बार पिलाया था। | Yes/ No  हॅा / नहीं |
| - 1. **Was baby roomed-in with the mother on Day 1?**   क्या बच्चे को पहले दिन मॅा के साथ एक कमरे में रखा था। | Yes/ No  हॅा / नहीं |
| - 1. **Was the mother counselled on breast feeding on Day 1 by hospital staff?**   क्या मॅा को किसी अस्पताल कर्मचारी ने पहले दिन मॅा का दूध कैसे पिलाना है, बताया था | Yes/ No  हॅा / नहीं |
| - 1. **When was the baby discharged? (Date and time)**   बेबी की डिस्चार्ज तारीख और समय |  |

1. **SCHEDULE FOR IN-DEPTH INTERVIEW (IDI)**

**साक्षातकार की सूची**

- 1. **Key instructions for the interviewer before starting the IDI**

Dear Interviewer,

- Please introduce each question verbatim, separately and record the response.
- If the respondent is unable to understand the question, repeat verbatim at a slower pace. If still unable to understand, ask which word is difficult to understand so that you could explain that in your own language.
- Please probe as suggested but do not prompt
- Please record the proceedings using the tape recorder/digital recorder with due labelling
- Please also record the verbatim responses in blank sheets provided for the question
- The elicited information is confidential

साक्षातकार लेने वाले के लिए अनुदेश

- क़ृपया सारे सवाल मौखिक पुछे और आवाज रिकार्ड करें।
- अगर प्रतिवादी को कोई सवाल समझने में कठिनाई हो, तो सवाल को धीरे से पूछे और जो शब्द उसे समक्ष नहीं आ रहे है उसे विस्तार से उसकी भाषा में बताएं।
- प्रतिवादी को स्वंय जवाब देने दे, बीच – बीच में आप उससे उचित बाते पूछे परन्तु उसे किसी जवाब के लिए प्ररेरित न करें।
- कृपया साक्षातकार को टेप रिकार्डर या डिजिटल रिकार्डर में रिकार्ड करे, लेबल के साथ।
- मौखिक प्रतिउत्तर को एक खाली पन्ने पर भी लिखित रूप से रिकार्ड करें।
- बताई गई सारी जानकारी गोपनीय रखी जाएगी।

**4.2 Introductory statement (To be read out aloud verbatim to the participant)**

*Namaskar!*

*Thank you for consenting to participate in this interaction. I am ____________________ from the Department of Neonatology, Ram Manohar Lohia Hospital, New Delhi. I am accompanied by ______________. This interaction will last for about 45 minutes. During this while, I will ask you some questions by reading out from a document. Please respond to these to the best of your capacity and interest. We will keep all the information confidential and you will not be identified in any of the project results. The information you share will be used only for research purpose in order to improve maternal and newborn care. Your facilitation and support are critical in this and we seek your valuable inputs for understanding the issues.*

*नमस्कार!*

*इस बातचीत के लिए समय निकलने के लिए बहुत बहुत शुक्रिया के लिए बहुत बहुत शुक्रिया. मैं डॉ .................... हूँ। यह साक्षात्कार लगभग ४५ मिनट्स चलेगा। इस दौरान मैं आपसे कुछ सवाल पूछूँगी/ पूछूंगा, एक पेपर से पढ़ कर कर। आपसे अनुरोध है की आप अपनी समझ और इच्छा के हिसाब से जवाब दें। हम यह सारी जानकारी अपने तक ही रखेंगे और किसी को भी नहीं बताएँगे । यह जानकारियां सिर्फ माँ और बच्चे को स्वास्थय लाभ पहुँचाने के लिए उपयोग की जाएँगी ।*

1. Questions (to be administered verbatim)
   1. Can you briefly describe the events that happened ever since you came to this hospital?

इस हस्पताल में आने के बाद से अबतक हुई चीज़ों के बारे में बताएं ?

- 1. Please share with us any advice that you have received on feeding your newborn at various points of your stay in the hospital and how has it influenced your practice? {Probe: advice from hospital: from whom, when and what advice was received (doctors, counsellors, nurses, others) advice from family members: from whom, when, what advice was received?}

आपको, बच्चे को दूध पिलाने के बारे मे अस्पताल में क्या क्या सलाह दी गयी और इसक| आपपे क्या असर पड़ा?

**(प्रोब:** अस्पताल से सलाह किसने, कब और क्या सलाह दी- डॉक्टर, नर्स, और/ अन्य कोई

परिवार वालों से मिली सलाह - किसने दी, कब दी और क्या सलाह दी

- 1. Please tell us about any challenges you have experienced regarding feeding your newborn **during your stay** in this hospital? Why do you feel this way? (Probes:
- skin-to-skin care and initiation of breastfeeding,
- feeding of colostrum and exclusive breastmilk feeding
- rooming-in
- sustaining the feeding of the newborn (as applicable): breastfeeding, expression of breast milk, formula feeding
- explore fear apprehensions in the context of corona; overall satisfaction; need for information/ communication

आप को अस्पताल में रहते हुए, अपने बच्चे को दूध पिलाने में क्या क्या परेशानिया हुई? आपको क्या लगता है यह परेशानिया क्यों हुई? **(प्रोब:**

- जनम के तुरंत बाद माँ की छाती से लगाया और पहली बार माँ का दूध कब पिलाया
- पहला गाढ़ा पीला दूध पिलाया/ केवल आपका ही दूध देना
- बच्चे को आपके साथ एक ही कमरे में रखा
- किस तरह से आप दूध पिलाते रही : जैसे छाती से पिलाया,या निकाल कर एक्सप्रेस्ड दूध पिलाया या फिर फार्मूला दूध दिया, या फिर कोई और तरीका अपनाया
- कोरोना के वजह से डर, चिंता, अस्पताल से जुडी संतुष्टि व कमियां, कोई और जानकारी चाहिए या फिर आपको लगा की किशी सलाह में कमी थी)
  1. According to you, how could these concerns be best addressed? How do you think this hospital and its staff could do better in helping you in feeding your newborn during your stay over here? (Probe: Once the participant speaks out full, ask her to reflect on the events around delivery and up to 48 hours thereafter, prevention of transmission of infection.)

आपने अभी जो बेबी को दूध पिलाने में हो रही दिक्कत्तों के बारे में बताया, उन सबको कैसे सही किया जा सकता है ? इसमें अस्पताल आपकी क्या और कैसे मदद कर सकता है ?

**(प्रोब:** जब माँ ने अपनी बात पूरी कर ली हो तब पूछे; डिलीवरी के समय और ४८ घंटो के अंदर उसके अस्पताल के अनुभव कैसे रहे; इन्फेक्शन से बचने के तरीके)

- 1. What things in this hospital you think have helped you in feeding your newborn? Why do you say so?

इस अस्पताल में आपको ऐसी कौनसी सुविधाएं मिली जिसकी वजह से आपके Baby को दूध पिलाने में आपको मदद हुई? आपको ऐसा क्यों लगता है ?

- 1. Given the corona situation, what kind of support do you, as a mother, expect for optimal feeding of your baby even after discharge and in follow up? How do you think this can be ensured? (Probe: role of family members and friends, hospital staff)

कोरोना के चलते, अस्पताल से छुट्टी के बाद, आपको तो अपने बेबी को दूध पिलाते रहना है . इसमें आपको मदद की ज़रुरत भी पड़ सकती है. आपने इसका कोई उपाय सोचा है क्या ? आप कैसे manage करोगी ? **(प्रोब:** परिवार से क्या मदद मिल सकती है? कोई दोस्त रिश्तेदार ? अस्पताल क्या मदद कर सकता है ?

- 1. Anything else you would want to add?

आप कुछ और बताना चाहेंगी?

Thank You

धन्यवाद

S3_COREQ (COnsolidated criteria for REporting Qualitative research) Checklist

A checklist of items that should be included in reports of qualitative research. You must report the page number in your manuscript where you consider each of the items listed in this checklist. If you have not included this information, either revise your manuscript accordingly before submitting or note N/A.

| **Topic** | **Item No.** | **Guide Questions/Description** | **Reported on**  **Page No.** |
| --- | --- | --- | --- |
| **Domain 1: Research team**  **and reﬂexivity** | | | |
| *Personal characteristics* | | | |
| Interviewer/facilitator | 1 | Which author/s conducted the interview or focus group? | **05 & 06** |
| Credentials | 2 | What were the researcher’s credentials? E.g. PhD, MD | **05 & 06** |
| Occupation | 3 | What was their occupation at the time of the study? | **05 & 06** |
| Gender | 4 | Was the researcher male or female? | **05 & 06** |
| Experience and training | 5 | What experience or training did the researcher have? | **05 & 06** |
| *Relationship with*  *participants* | | | |
| Relationship established | 6 | Was a relationship established prior to study commencement? | **06** |
| Participant knowledge of  the interviewer | 7 | What did the participants know about the researcher? e.g. personal  goals, reasons for doing the research |  |
|  |  |  | **06** |
|  |  |  |  |
| Interviewer characteristics | 8 | What characteristics were reported about the interviewer/facilitator?  e.g. Bias, assumptions, reasons and interests in the research topic |  |
|  |  |  | **06** |
|  |  |  |  |
| **Domain 2: Study design** | | | |
| *Theoretical framework* | | | |
| Methodological orientation and Theory | 9 | What methodological orientation was stated to underpin the study? e.g. grounded theory, discourse analysis, ethnography, phenomenology,  content analysis |  |
|  |  |  | **04** |
|  |  |  |  |
| *Participant selection* | | | |
| Sampling | 10 | How were participants selected? e.g. purposive, convenience,  consecutive, snowball |  |
|  |  |  | **05** |
|  |  |  |  |
| Method of approach | 11 | How were participants approached? e.g. face-to-face, telephone, mail,  email |  |
|  |  |  | **05** |
|  |  |  |  |
| Sample size | 12 | How many participants were in the study? | **07** |
| Non-participation | 13 | How many people refused to participate or dropped out? Reasons? | **07** |
| *Setting* | | | |
| Setting of data collection | 14 | Where was the data collected? e.g. home, clinic, workplace | **06** |
| Presence of non-  participants | 15 | Was anyone else present besides the participants and researchers? |  |
|  |  |  | **06** |
|  |  |  |  |
| Description of sample | 16 | What are the important characteristics of the sample? e.g. demographic  data, date |  |
|  |  |  | **05** |
|  |  |  |  |
| *Data collection* | | | |
| Interview guide | 17 | Were questions, prompts, guides provided by the authors? Was it pilot  tested? | **05** |
|  |  |  |  |
| Repeat interviews | 18 | Were repeat inter views carried out? If yes, how many? | **N.A.** |
| Audio/visual recording | 19 | Did the research use audio or visual recording to collect the data? | **06 & 07** |
| Field notes | 20 | Were ﬁeld notes made during and/or after the interview or focus group? | **06** |
| Duration | 21 | What was the duration of the inter views or focus group? | **07** |
| Data saturation | 22 | Was data saturation discussed? | **06** |
| Transcripts returned | 23 | Were transcripts returned to participants for comment and/or | **N.A.** |

| **Topic** | **Item No.** | **Guide Questions/Description** | **Reported on**  **Page No.** |
| --- | --- | --- | --- |
|  |  | correction? |  |
| **Domain 3: analysis and**  **ﬁndings** | | | |
| *Data analysis* | | | |
| Number of data coders | 24 | How many data coders coded the data? | **07** |
| Description of the coding  tree | 25 | Did authors provide a description of the coding tree? |  |
|  |  |  | **08** |
| Derivation of themes | 26 | Were themes identiﬁed in advance or derived from the data? | **07** |
| Software | 27 | What software, if applicable, was used to manage the data? | **07** |
| Participant checking | 28 | Did participants provide feedback on the ﬁndings? | **08** |
| *Reporting* | | | |
| Quotations presented | 29 | Were participant quotations presented to illustrate the themes/ﬁndings?  Was each quotation identiﬁed? e.g. participant number |  |
|  |  |  | **08** |
|  |  |  |  |
| Data and ﬁndings consistent | 30 | Was there consistency between the data presented and the ﬁndings? | **08** |
| Clarity of major themes | 31 | Were major themes clearly presented in the ﬁndings? | **09** |
| Clarity of minor themes | 32 | Is there a description of diverse cases or discussion of minor themes? | **09** |

Developed from: Tong A, Sainsbury P, Craig J. Consolidated criteria for reporting qualitative research (COREQ): a 32-item checklist for interviews and focus groups. *International Journal for Quality in Health Care*. 2007. Volume 19, Number 6: pp. 349 – 357

**Once you have completed this checklist, please save a copy and upload it as part of your submission. DO NOT include this checklist as part of the main manuscript document. It must be uploaded as a separate file.**
